# Synthesis of chiral mono(N-heterocyclic carbene) palladium and gold complexes with a 1,1'-biphenyl scaffold and their applications in catalysis

**DOI:** 10.3762/bjoc.7.64

**Published:** 2011-05-04

**Authors:** Lian-jun Liu, Feijun Wang, Wenfeng Wang, Mei-xin Zhao, Min Shi

**Affiliations:** 1 Key Laboratory for Advanced Materials and Institute of Fine Chemicals, School of Chemistry & Molecular Engineering, East China University of Science and Technology, 130 MeiLong Road, Shanghai 200237, People’s Republic of China; 2 State Key Laboratory of Organometallic Chemistry, Shanghai Institute of Organic Chemistry, Chinese Academy of Sciences, 354 Fenglin Road, Shanghai 200032, People’s Republic of China, Fax: 86-21-64166128

**Keywords:** chiral mono(N-heterocyclic carbene) complex, Heck–Mizoroki reaction, hydroamination, Suzuki–Miyaura reaction

## Abstract

Axially chiral mono(NHC)–Pd(II) and mono(NHC)–Au(I) complexes with one side shaped 1,1'-biphenyl backbone have been prepared from chiral 6,6'-dimethoxybiphenyl-2,2'-diamine. The complexes were characterized by X-ray crystal structure diffraction. The Pd(II) complex showed good catalytic activities in the Suzuki–Miyaura and Heck–Mizoroki coupling reactions, and the (*S*)-Au(I) complexes also showed good catalytic activities in the asymmetric intramolecular hydroamination reaction to give the corresponding product in moderate ee.

## Introduction

**Figure 1:**
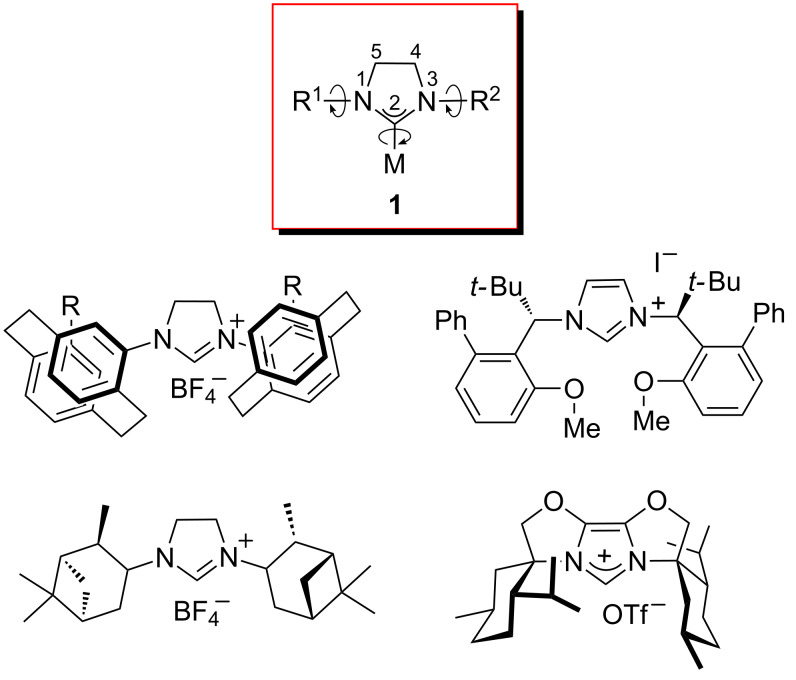
Monodentate NHCs with sterically hindered N-substituents.

N-heterocyclic carbene (NHC) ligands, which have intrinsic characteristics such as strong σ-donor but poor π-acceptor abilities, easy preparation, air and thermal stability of their metal complexes, and convenient introduction of chiral elements, have been widely used as promising ligands in metal-catalyzed transformations [1–13]. Numbers of novel chiral NHCs and NHC–metal-catalyzed asymmetric transformations have been developed in a dramatic expansion of this area of chemistry during the past decade; however, up to 2010 only a very few efficient chiral NHCs or NHCs metal catalysts have been described [14–18]. From the typical configuration of NHC metal complex **1** (Figure 1), NHCs are generally more or less cone-shaped with flat heterocyclic structures, and that R^1^, R^2^ and M can rotate flexibly around the R^1^–N, R^2^–N and C–M bonds, respectively. Such internal rotations cause the active chiral space at the metal center to be relatively ill-defined, which is a key factor for their low enantioselectivity in asymmetric catalysis. As a result, many monodentate NHCs (Figure 1) with sterically hindered R^1^, R^2^ groups have been designed, and these have been shown to be good to excellent catalysts in chiral induction reactions [19–25].

**Figure 2:**
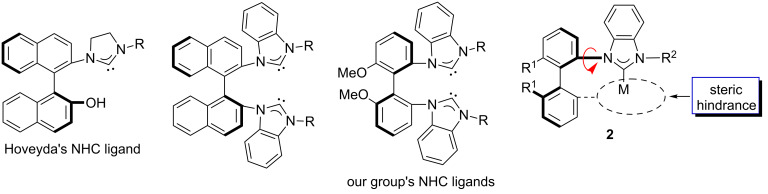
NHCs with axially chiral biaryl frameworks.

The axially chiral biaryl framework, widely used in the design of chiral ligands such as BINAP [26–27], BINOL [28–29], and boxax [30], has proved to be very rigid, and was introduced in the development of NHC ligands by the Hoveyda group [31–36] and ours [37–44] (Figure 2). Several highly efficient asymmetric catalytic processes with these novel chiral NHCs-bonded metal catalysts have so far been reported. Encouraged by these results, we attempted to develop a new type of mono(NHC) metal complex **2** with a biaryl framework, in which one of biaryl groups bearing a substituent might provide steric hindrance to limit the rotation of the N–Ar bond. Herein we wish to report the synthesis of novel chiral [(NHC)Pd(allyl)I] and mono(NHC)–Au complexes bearing an axially chiral biphenyl framework, and their application in catalysis.

## Results and Discussion

### 

#### Synthesis of the NHC–Pd(II) and NHC–Au(I) complexes

**Scheme 1:**
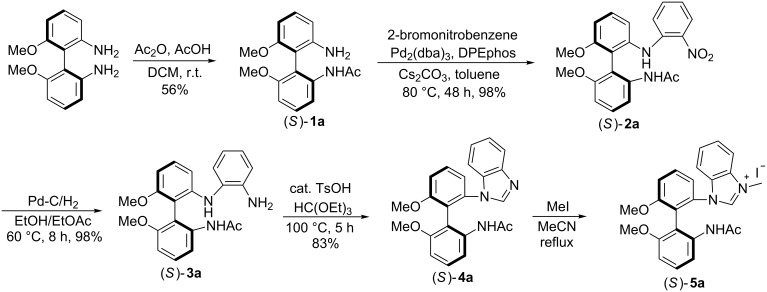
Synthesis of N-heterocyclic carbene precursor.

The synthesis of the chiral benzimidazolium salt (*S*)-**5a** is shown in Scheme 1. Thus (*S*)-6,6'-dimethoxybiphenyl-2,2'-diamine was reacted with acetic anhydride in the presence of acetic acid at room temperature (25 °C) in DCM to afford the corresponding amide (*S*)-**1a** in 56% yield. The coupling reaction of (*S*)-**1a** with 2-bromonitrobenzene was achieved by the use of bis(2-diphenylphosphinophenyl)ether (DPEphos) as a ligand and Pd_2_(dba)_3_ as the catalyst in the presence of Cs_2_CO_3_ to give the desired compound (*S*)-**2a** in 98% yield. Reduction of (*S*)-**2a** by means of Pd-C/H_2_ for 8 h gave (*S*)-**3a** in 98% yield. Subsequent cyclization with triethyl orthoformate catalyzed by *p*-toluenesulfonic acid at 100 °C for 5 h afforded (*S*)-**4a** in 83% yield. Quaternization of the benzimidazole ring of (*S*)-**4a** by heating with methyl iodide in acetonitrile provided the corresponding benzimidazolium salt (*S*)-**5a** in quantitative yield.

**Scheme 2:**
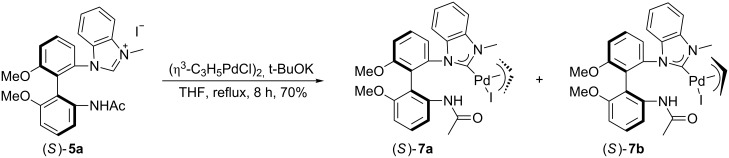
Synthesis of mono(NHC)–Pd(II) complex.

**Figure 3:**
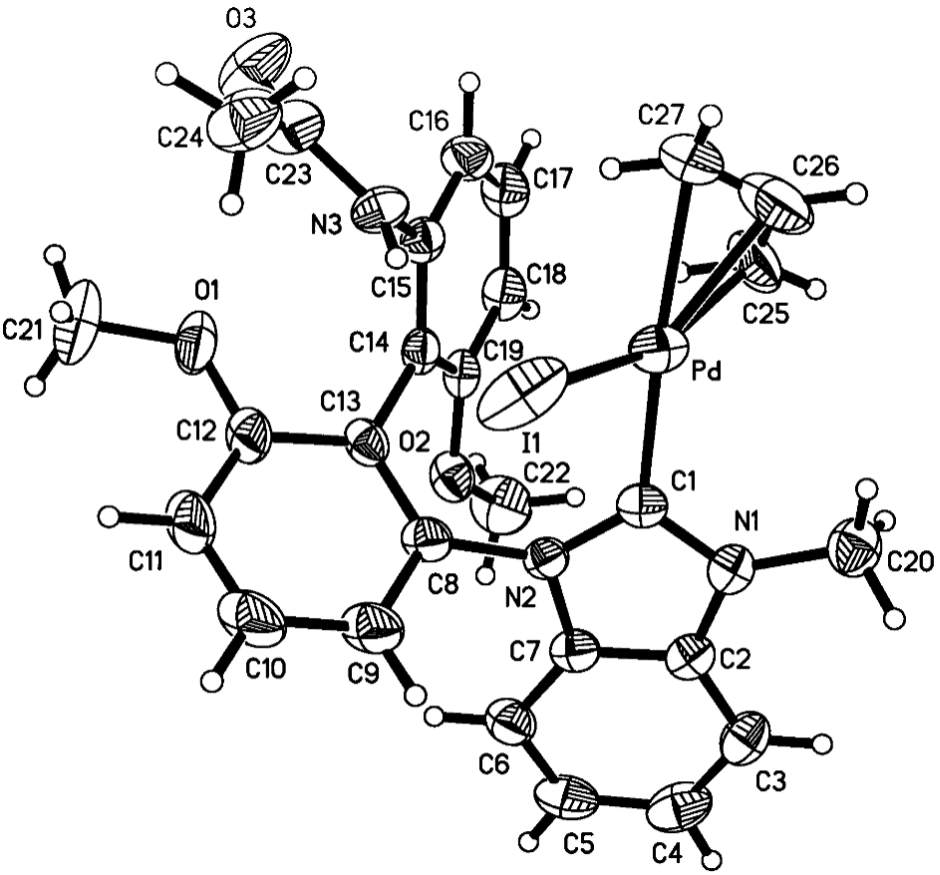
ORTEP drawing of NHC–Pd(II) complex (*S*)-**7a** with thermal ellipsoids at the 30% probability level. Selected bond distances (Å) and angles (deg): Pd–C1 = 2.050(5), Pd–C25 = 2.154(7), Pd–C26 = 2.128(9), Pd–C27 = 2.168(8), Pd–I1 = 2.6404(7), N1–C1–Pd = 125.6(4), N2–C1–Pd = 128.8(4), C1–Pd–I1 = 99.52(13), C1–Pd–C25 = 98.3(3), C1–Pd–C26 = 132.3(5), C1–Pd–C27 = 163.7(4), I1–Pd–C25 = 161.9(3), I1–Pd–C26 = 126.5(5), I1–Pd–C27 = 95.9(3), C25–Pd–C27 = 66.0(4), C25–Pd–C26 = 37.5(4), C26–Pd–C27 = 34.8(4).

With the NHC precursor (*S*)-**5a** in hand, its coordination with Pd or Au metal salts was examined. Benzimidazolium salt (*S*)-**5a** was treated with (η^3^-C_3_H_5_PdCl)_2_ in tetrahydrofuran (THF) in the presence of *t*-BuOK at 50 °C to give [(NHC)Pd(allyl)I] complex (*S*)-**7** in 70% yield as a yellow solid after purification by silica gel column chromatography (Scheme 2) [45–46]. Due to the stereochemical orientation of π-allyl group relative to the unsymmetrical carbene ligand, this NHC–Pd complex exists as two stereoisomers in solution, (*S*)-**7a** and (*S*)-**7b**, which could be easily distinguished in its ^1^H NMR spectrum recorded at 23 °C [47–50]. The ratio of (*S*)-**7a** and (*S*)-**7b** was found to be 1.2:1 on the basis of ^1^H NMR spectroscopic data. It appears that (*S*)-**7a** is slightly more stable than (*S*)-**7b** presumably due to the steric repulsion between the π-allyl group and the acetylated amino group in another phenyl group. However, we were unable to isolate either stereoisomer in a pure form by silica gel column chromatography. After recrystallization from DCM and pentane (1:3), one of the two stereoisomers, [(NHC)Pd(allyl)I] (*S*)-**7a**, was obtained as a crystalline compound and its structure was confirmed by the X-ray single crystal diffraction (Figure 3) [51].

**Scheme 3:**
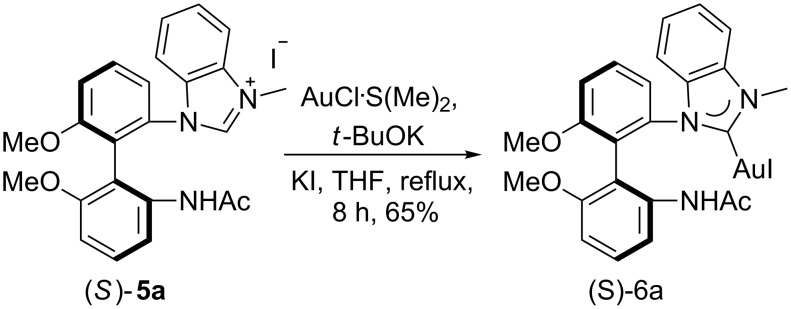
Synthesis of mono(NHC)–Au(I) complex.

**Figure 4:**
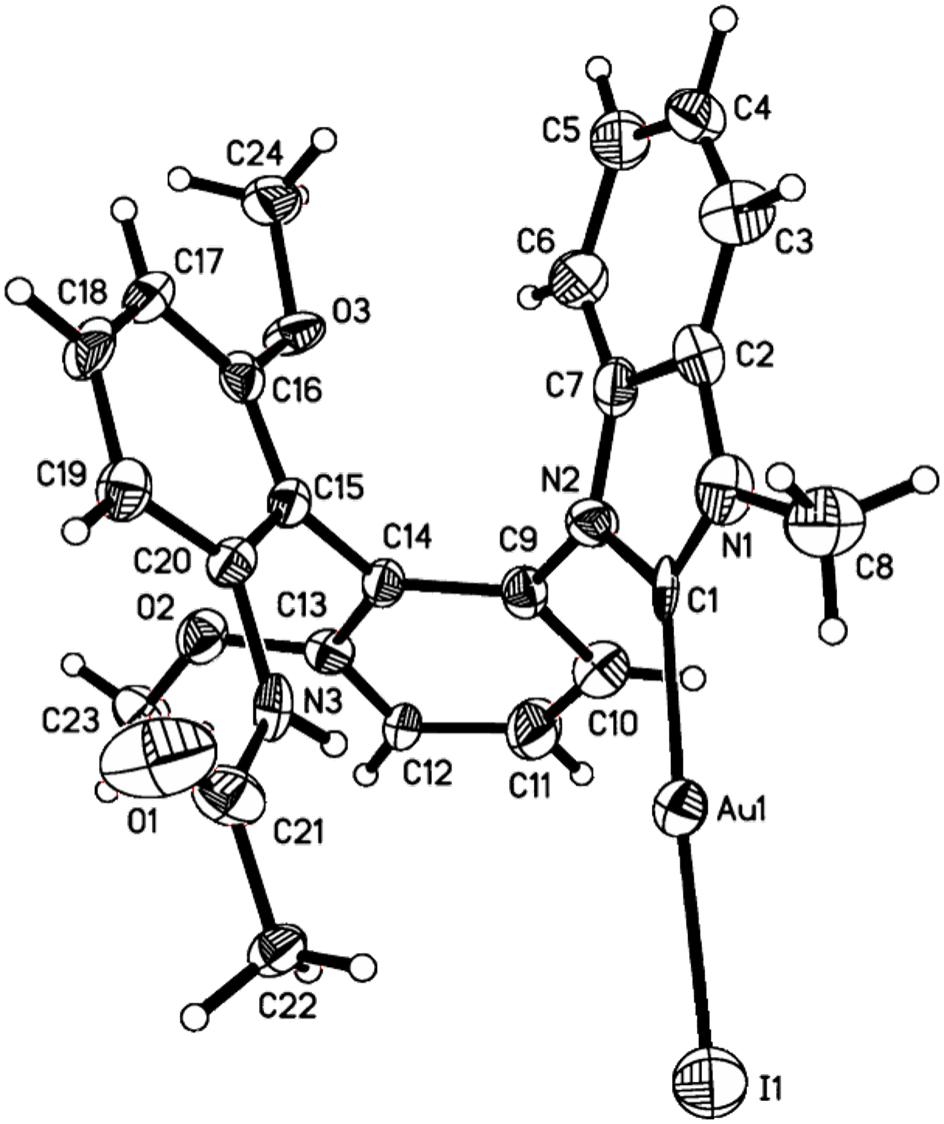
ORTEP drawing of NHC–Au(I) complex (*S*)-**6a** with thermal ellipsoids at the 30% probability level. Selected bond distances (Å) and angles (deg): Au1–C1 = 2.036(14), Au1–I1 = 2.5093(13), C1–Au1–I1 = 176.5(3), N1–C1–Au1 = 125.7(9), N2–C1–Au = 126.1(9).

Benzimidazolium salt (*S*)-**5a** also complexed Au(I). According to the previously reported procedure [52–55], (*S*)-**5a** reacted with AuCl·S(Me) _2_ on heating in THF for 8 h in the presence of KI and *t*-BuOK to give the expected chiral NHC–Au(I) complex (*S*)-**6a** in 65% yield (Scheme 3). Its structure was also confirmed by X-ray diffraction (Figure 4) [56]. It was found that the Au–carbene distance is 2.036 Å which is consistent with other reported NHC–Au complexes [57–59].

The NHC–metal complex [(NHC)Pd(allyl)I] complex **7** and NHC–Au(I) complex (*S*)-**6a**, are air and moisture stable both in the solid state and in solution, and can be used as catalysts. Therefore, the catalytic activities of these complexes were investigated in Pd-catalyzed coupling reactions and a Au-catalyzed asymmetric reaction, respectively.

#### Suzuki–Miyaura and Heck–Mizoroki coupling reactions catalyzed by NHC–Pd(II) complex

The Pd-catalyzed coupling reaction is one of the most powerful methods for the formation of carbon–carbon bonds in organic synthesis [60–66]. NHC–Allylpalladium complexes have been employed and showed good catalytic activities in carbon–carbon bond coupling reactions [67–70]. Stereoisomeric complex **7** was firstly applied as the catalyst in the catalyze Suzuki–Miyaura coupling reaction. On the basis of screening of the solvent and base in the reaction of phenylboronic acid with bromobenzene, it was found that using *t*-BuOK as the base in iPrOH at 50 °C for 10 h, the coupling product **9a** was obtained in the highest yield (81% yield) (Supporting Information File 1). Under the optimized conditions, the reactions of various aryl halides with arylboronic acids were carried out, and it was found that the electronic properties of the R groups and halide atoms significantly affected the reaction yield of the Suzuki–Miyaura reactions. The results have been summarized in Table 1.

**Table 1:** Suzuki–Miyaura reaction catalyzed by NHC–Pd(II) complex **7**^a^.

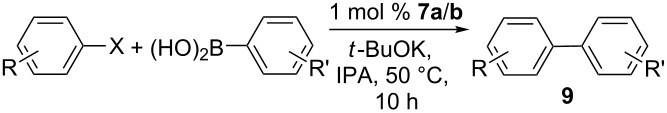					

Entry	R	R'	X	Product	Yield (%)^b^

1	4-Me	H	Br	**9a**	81
2	4-COMe	H	Br	**9b**	98
3	2-NO_2_	H	Br	**9c**	96
4	2-MeO	H	Br	**9d**	89
5	2-Me	H	Br	**9e**	80
6	4-COMe	4-Me	Br	**9f**	97
7	4-MeO	4-Me	Br	**9g**	81
8	4-Me	2-Cl	Br	**9h**	74
9	H	4-Me	I	**9a**	>99
10	H	4-Me	Cl	**9a**	<5

^a^Reaction conditions: 1 mmol aryl halide, 1.3 mmol arylboronic acid, 1.3 mmol *t*-BuOK, 0.01 mmol NHC–Pd(II) complex, 2.0 mL IPA. ^b^Isolated yields.

The [(NHC)Pd(allyl)I] complex **7** was also examined in the Heck–Mizoroki coupling reaction. Under optimized conditions (Supporting Information File 1), the reactions of various aryl halides with *n*-butyl acrylate were carried out in the presence of Na_2_CO_3_ in *N*,*N*-dimethylacetamide (DMA) at 140 °C. [(NHC)Pd(allyl)I] complex **7** showed good catalytic activities in the reaction of arylbromides or iodobenzene with *n*-butyl acrylate to afford the coupling products **10** in up to 97% yield. The results have been summarized in Table 2.

**Table 2:** Heck–Mizoroki reaction catalyzed by NHC–Pd(II) complex **7**^a^.

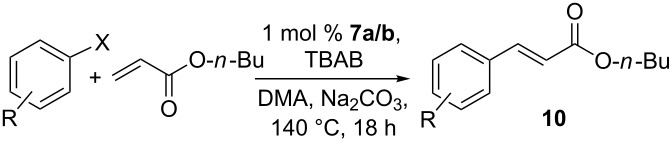					

Entry	R	X	Time (h)	Product	Yield (%)^b^

1	H	Br	18	**10a**	82
2	4-Me	Br	18	**10b**	79
3	4-MeO	Br	18	**10c**	80
4	4-CHO	Br	18	**10d**	85
5	2-Me	Br	18	**10e**	76
6	H	I	18	**10a**	97
7	H	Cl	18	**10a**	<5

^a^Reaction conditions: **7** (1 mol % Pd), Na_2_CO_3_ (2.0 mmol), TBAB (0.1 mmol), aryl halide (1.0 mmol) and *n*-butyl acrylate (1.5 mmol) in DMA (3.0 mL) at 140 °C for 18 h. ^b^Isolated yield after silica gel column chromatography.

#### Intramolecular hydroamination reaction catalyzed by NHC–Au(I) complex (*S*)-6a

Since the first example of NHC–Au complex was reported in 1989 [71], a variety of neutral or cationic NHC–Au complexes has been synthesized and applied in many catalytic reactions [72–73]. For example, recently, NHC–Au showed good catalytic activity in the intramolecular [4 + 2] cycloadditions of 1,3-enynes or arylalkynes [74], rearrangement of allylic acetates [75–76], carbene-transfer reactions from ethyl diazoacetate [77], formation of conjugated enones and enals [78], regio- and stereoselective synthesis of fluoroalkenes [79], and so on [80–85]. However, reports on NHC–Au catalyzed asymmetric reactions are rare [86]. The NHC–Au(I) complex (*S*)-**6a** was consequently investigated as the catalyst in the asymmetric intramolecular hydroamination of allenes. This reaction has been achieved with high enantioselectivity by a chiral phosphine–Au(I) complex [87–93]. Treatment of allene **11** with (*S*)-**6a** and AgSbF_6_ (5 mol %) in DCM at room temperature for 36 h afforded pyrrolidine derivative **12** in 53% yield with an ee of only 10%. When THF or toluene was used as solvent, only traces of compound **12** were formed. Further screening of AgX revealed that the combination of (*S*)-**6a** and AgClO_4_ gave the best catalytic activity in this reaction (Table 3).

**Table 3:** NHC–Au complex (*S*)-**6a** catalyzed asymmetric intramolecular hydroamination.

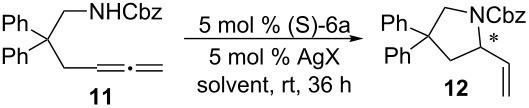					

Entry	Solvent	AgX	Time (h)	Yield (%)^a^	ee (%)^b^

1	THF	AgSbF_6_	36	trace	—^c^
2	Toluene	AgSbF_6_	36	trace	—
3	DCM	AgSbF_6_	36	53	10
4	DCE	AgSbF_6_	36	43	7
5	DCM	AgClO_4_	36	63	10
6	DCM	AgOTf	36	42	10
7	DCM	AgOTs	36	—^d^	—
8	DCM	AgBF_4_	36	46	0

^a^Isolated yield. ^b^Determined by chiral HPLC. ^c^not determined. ^d^no reaction.

We assumed that the ill-defined chiral space at the Au center may be the cause of the low ee, and that a more sterically bulky group than an acetyl group in another phenyl framework may be required to improve the enantioselectivity. Accordingly, the original acetyl group was replaced by a more sterically bulky group such as *tert*-butoxycarbonyl group and adamantanecarbonyl group.

#### Synthesis of the NHC–Au(I) complexes (*S*)-6b and 6c

**Scheme 4:**
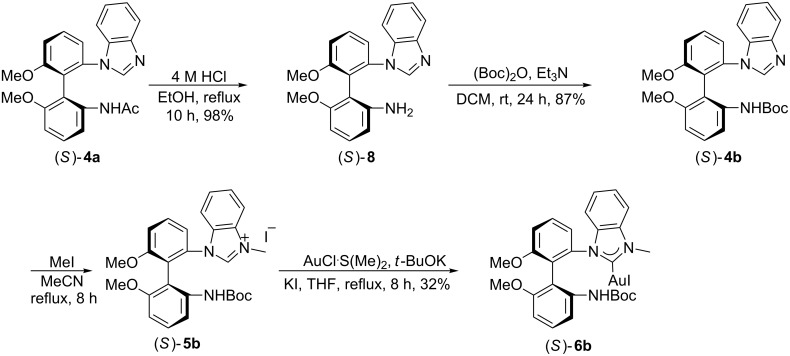
Synthesis of mono(NHC)–Au(I) complex (*S*)-**6b**.

The synthesis of (*S*)-**6b** is shown in Scheme 4: Thus (*S*)-**4a** was heated under refux with 4 M HCl in EtOH to afford the corresponding amide (*S*)-**8** in 98% yield. Amine (*S*)-**8** was then treated with (Boc)_2_O in the presence of Et_3_N at room temperature for 24 h to give the corresponding BOC derivative (*S*)-**4b** in 87% yield. Quaternization of the benzimidazole ring of (*S*)-**4b** with methyl iodide in acetonitrile gave the corresponding benzimidazolium salt (*S*)-**5b** in quantitative yield. Benzimidazolium salt (*S*)-**5b** was then complexed with Au (I) as described above for (*S*)-**6a** (AuCl·S(Me)_2_ in the presence of KI and *t*-BuOK in THF for 8 h) to produce the expected chiral NHC–Au(I) complex (*S*)-**6b** in 32% yield.

**Scheme 5:**
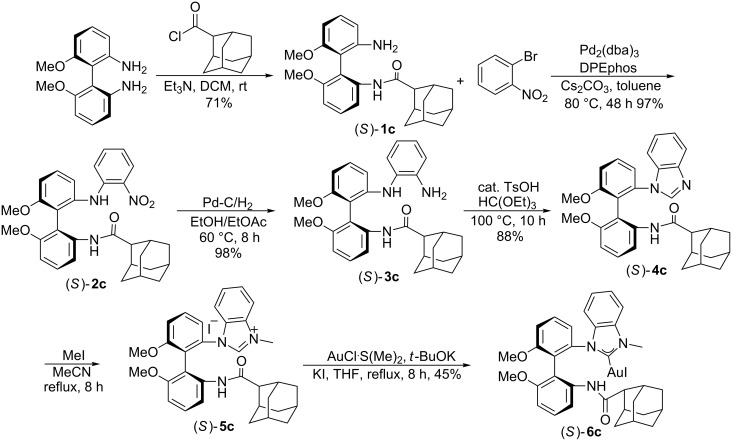
Synthesis of mono(NHC)–Au(I) complex (*S*)-**6c**.

For the preparation of (*S*)-**6c**, (*S*)-6,6'-dimethoxybiphenyl-2,2'-diamine was treated with adamantane-2-carbonyl chloride in the presence of Et_3_N at room temperature (25 °C) in DCM to afford the corresponding amide (*S*)-**1c** in 71% yield. According to the synthetic method for the synthesis of compound (*S*)-**6a**, NHC–Au complex (*S*)-**6c** was successfully prepared in 45% yield (Scheme 5).

**Scheme 6:**
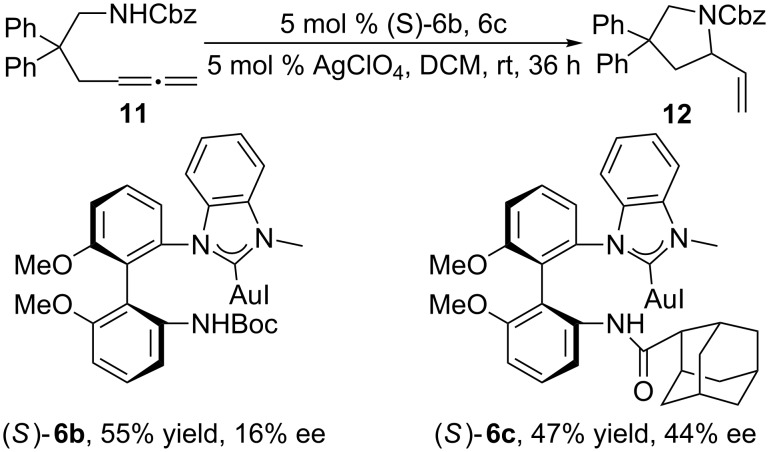
The application of catalysts (*S*)-**6b** and **6c** in the intramolecular hydroamination reaction.

Under the optimized conditions, (*S*)-**6b** and (*S*)-**6c** were used as catalysts to examine their chiral induction abilities in the intramolecular hydroamination reaction (Scheme 6). As expected, the corresponding pyrrolidine derivative **12** was obtained in higher ee value but in lower isolated yield: With (*S*)-**6c** as catalyst, **12** was obtained in 47% yield with an ee of 44% whereas with (*S*)-**6b** as catalyst, **12** was obtained in 55% yield but the ee was only 16%.

## Conclusion

Axially chiral mono(NHC)–Pd(II) and mono(NHC)–Au(I) complexes with one side shaped 1,1'-biphenyl backbone have been prepared from chiral 6,6'-dimethoxybiphenyl-2,2'-diamine. The Pd(II) complex showed good catalytic activity in the Suzuki–Miyaura and Heck–Mizoroki coupling reactions. The (*S*)-Au(I) complex also showed moderate catalytic activities along with moderate chiral inductions in the asymmetric intramolecular hydroamination reaction. Using chiral Au complex (*S*)-**6c**, having a sterically hindered 2-adamantanecarbonyl group, as the catalyst gave the corresponding intramolecular hydroamination product in higher enantioselectivity (44% ee). Efforts are underway to extend the scope and limitations of these chiral (NHC) Pd(II) and Au(I) complexes in other asymmetric catalytic reactions.

## Experimental

### 

#### Synthesis of NHC–Pd(II) complex **7**

Compound **5a** (105.8 mg, 0.2 mmol) and [PdCl(η^3^-allyl)]_2_ (109.1 mg, 0.3 mmol), *t*-BuOK (56 mg, 0.5 mmol) were heated under reflux in THF (10 mL) for 8 h. The volatiles were then removed under reduced pressure and the residue purified by a silica gel flash column chromatography (eluent: petroleum ether/ethyl acetate, 2:1–0:1) to give **7** as a mixture of two isomers (117.0 mg, 70%). A single crystal grown from racemic complex **7** in a saturated solution of DCM:pentane (1/3) was suitable for X-ray crystal structure analysis. (*S*)-**7**, light yellow solid; mp: 124.6–125.3 °C; [α]_D_^20^ +13.0 (*c* 0.25, CHCl_3_); IR (DCM) ν 3303, 3037, 2933, 2838, 1688, 1688, 1594, 1520, 1464, 1378, 1256, 1125, 1090, 1062, 1004, 976, 939, 733, 560, 530 cm^−1^; ^1^H NMR (400 MHz, CDCl_3_, TMS): δ [2.07 (s, CH_3_), 2.14 (s, CH_3_), 1:1.2, 3H], [2.53 (d, *J* = 12.0 Hz, CH_2_), 2.71 (d, *J* = 13.2 Hz, CH_2_), 1:1.2, 1H], [2.84 (s, OCH_3_), 2.88 (s, OCH_3_), 1:1.2, 3H], [3.07 (d, *J* = 13.2. Hz, CH_2_), 3.65 (d, *J* = 6.8 Hz, CH_2_), 1.2:1, 1H], [3.76 (s, OCH_3_), 3.79 (s, OCH_3_), 1:1.2, 3H], [3.81 (s, CH_3_), 3.92 (s, CH_3_), 1:1.2, 3H], 4.23 (brs, 1H, CH_2_), [4.86–4.95 (m, CH), 5.19–5.29 (m, CH), 1:1.2, 1H], [6.35 (d, *J* = 8.4 Hz, CH_2_), 6.39 (d, *J* = 8.0. Hz, CH_2_), 1:1.2, 1H], 7.09–7.23 (m, 3H, ArH), 7.29–7.36 (m, 4H, ArH), 7.43–7.69 (m, 3H, ArH), [8.10 (s, NH), 8.18 (s, NH), 1:1.2, 1H]; MS (ESI) *m/z* (%): 675 (M^+^, 60.07), 402 (M^+^−273, 100), 274 (M^+^−401, 28.80); Anal. Calcd. for C_27_H_28_IN_3_O_3_Pd requires: C, 47.98; H, 4.18; N, 6.22%. Found: C_27_H_28_IN_3_O_3_Pd, C 47.78, H 4.68, N 5.78%.

#### Synthesis of NHC–Au(I) complex (S)-**6a**

Compound (*S*)-**5a** (105.8 mg, 0.2 mmol) and AuCl·S(Me)_2_ (58.8 mg, 0.2 mmol), KI (49.8 mg, 0.3 mmol) *t*-BuOK (56 mg, 0.5 mmol) were heated under reflux in THF (10 mL) for 8 h. The volatiles were then removed under reduced pressure and the residue purified by a silica gel flash column chromatography (eluent: petroleum ether/ethyl acetate, 2:1–0:1) to give **8** as a white solid (94 mg, 65%). A single crystal grown from racemic complex **6a** in a saturated solution of DCM/pentane (1:3) was suitable for X-ray crystal structure analysis. (*S*)-**6a**: white solid; mp: 184.3–129.6 °C; [α]_D_^20^ +5.0 (*c* 0.25, CHCl_3_); IR (DCM) ν 3407, 2929, 2835, 1697, 1591, 1468, 1438, 1286, 1083, 1002, 852, 779, 747, 657 cm^−1^; ^1^H NMR (400 MHz, CDCl_3_, TMS): δ 2.19 (s, CH_3_, 3H), 3.21 (s, OCH_3_, 3H), 3.81 (s, OCH_3_, 3H), 3.97 (s, CH_3_, 3H), 6.36 (d, *J* = 8.4 Hz, Ar, 1H), 7.11–7.25 (m, Ar and NH, 5H), 7.32–7.37 (m, Ar, 4H), 7.47 (d, *J* = 8.0 Hz, Ar, 1H), 7.62 (t, *J* = 8.0 Hz, Ar, 1H); MS (ESI) *m/z* (%): 551 (M^+^, 10.05), 598 (M^+^−127, 100), 612 (M^+^−113, 22.10); Anal. Calcd. for C_24_H_23_IN_3_O_3_Au requires: C, 39.74; H, 3.20; N, 5.79%. Found: C_24_H_23_IN_3_O_3_Au C 40.64, H 3.08, N 5.72%.

#### General procedure for the intramolecular hydroamination reaction catalyzed by NHC–Au(I) complex (*S*)-**6a**

A mixture of NHC–Au(I) (*S*)-**6a** (7.2 mg, 5 mol %) and AgX (5 mol %) in DCM (0.4 mL) was stirred at rt for 5 min. A solution of compound **11** (76.6 mg, 0.20 mmol) in DCM (0.6 mL) was added to the resulting solution and the mixture stirred at rt for 36 h. Column chromatography of the reaction mixture gave the desired product. The enantiomeric purity of the product was determined by chiral HPLC analysis.

## Supporting Information

File 1 Experimental procedures and characterization data of compounds given in this article.

File 2 Crystal structure data for NHC–Pd(II) complex **7a**.

File 3 Crystal structure data for NHC–Au(I) complex **6a**.

File 4 Crystal structure information file of compound **6a**.

File 5 Crystal structure information file of compound **7a**.
