# Supplementary material for: Synthesis of chiral mono(N-heterocyclic carbene) palladium and gold complexes with a 1,1'-biphenyl scaffold and their applications in catalysis
Source: Beilstein J Org Chem. 2011 May 4;7:555–64. doi: 10.3762/bjoc.7.64 (PMC3107448; doi:10.3762/bjoc.7.64)
Supplement: File 1 — Experimental procedures and characterization data of compounds given in this article. [file Beilstein_J_Org_Chem-07-555-s001.pdf]

Supporting Information

for

Synthesis of chiral mono(N-heterocyclic carbene) palladium and gold complexes with a 1,1'-biphenyl scaffold and their applications in catalysis

Lian-jun Liu[1], Feijun Wang[1], Wenfeng Wang[1], Mei-xin Zhao[1] and Min Shi*[1,2]

Address: [1]Key Laboratory for Advanced Materials and Institute of Fine Chemicals, School of Chemistry & Molecular Engineering, East China University of Science and Technology, 130 MeiLong Road, Shanghai 200237, People's Republic of China and [2]State Key Laboratory of Organometallic Chemistry, Shanghai Institute of Organic Chemistry, Chinese Academy of Sciences, 354 Fenglin Road, Shanghai 200032, People's Republic of China, Fax: 86-21-64166128

Email: Min Shi* - Mshi@mail.sioc.ac.cn

*Corresponding author

Experimental procedures and characterization data of compounds given in this article

CONTENTS

General remarks S2

Synthesis of NHC-Pd(II) complex **7** and NHC-Au(I) complex (*S*)-**6a** S2

The synthesis of NHC-Pd complexes (*S*)-**7** S9

The synthesis of (*S*)-**8** S11

General procedure for the NHC-Pd complex-catalyzed Suzuki–Miyaura cross-coupling reaction of aryl bromides with phenylboronic acids S24

General procedure for the Heck–Mizoroki cross-coupling reaction of aryl halide with *n*-butyl acrylate S26

General procedure for the intramolecular hydroamination reaction catalyzed by NHC-Au(I) complex (*S*)-**6a** S27

References S28

General remarks. Dichloromethane was freshly distilled from calcium hydride; THF and toluene were distilled from sodium under an argon atmosphere. Melting points were determined on a digital melting point apparatus and temperatures and are uncorrected. ^{1}H NMR and ^{13}C NMR spectra were recorded on a Bruker AM-400 spectrometer. Infrared spectra were recorded on a Perkin-Elmer PE-983 spectrophotometer with absorptions in cm^{-1}. Flash column chromatography was performed on 300–400 mesh silica gel. For thin-layer chromatography (TLC), silica gel plates (Huanghai GF254) were used. Elementary analysis was taken on a Carlo-Erba 1106 analyzer. Mass spectra were recorded by ESI, and HRMS were measured on a HP-5989 instrument.

Synthesis of NHC–Pd(II) complex 7 and NHC–Au(I) complex (*S*)-6a

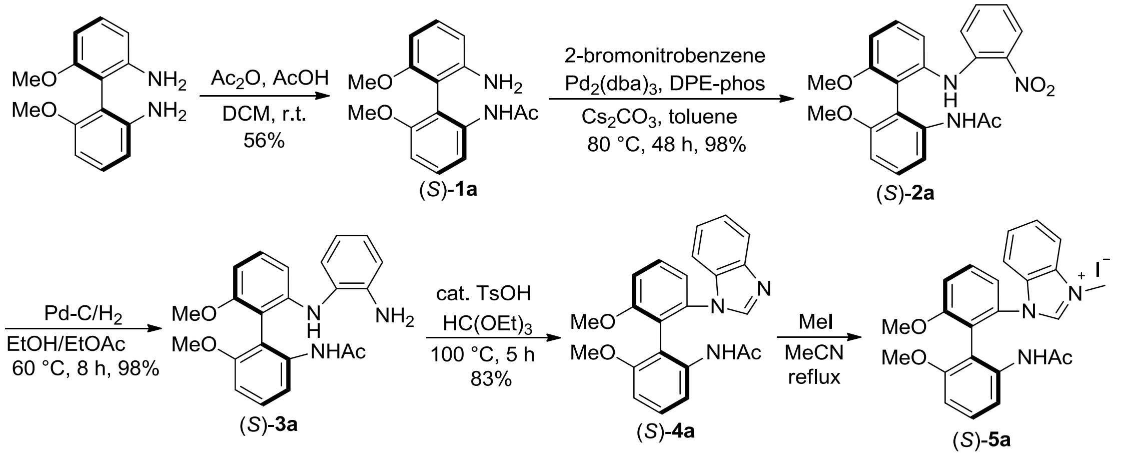

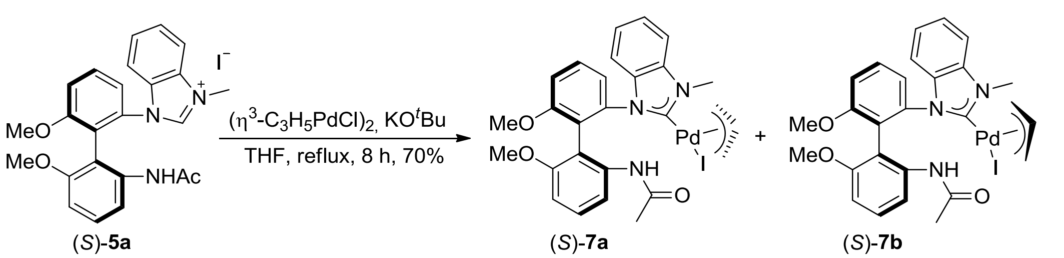

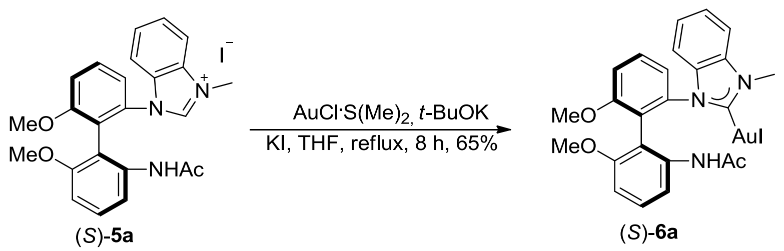

Synthesis of compound (*S*)-1a

6,6'-Dimethoxybiphenyl-2,2'-diamine (244.1 mg, 1.0 mmol) and AcOH (0.6 mL, 10 mmol) in 10 mL of dried CH_2Cl_2 was treated with acetic anhydride (104 μL, 1.0 mmol) at 0 °C, and the resulting solution stirred overnight at room temperature. Aqueous NaOH solution (2.0 N) was added to adjust the pH to ≈ 7. The reaction mixture was extracted by CH_2Cl_2 (3 x 50 mL) and the combined organic phases were washed with saturated brine and dried over $MgSO_4$. The solvent was removed under reduced pressure and the crude product was purified by flash chromatography (ethyl acetate/hexane = 2/1) to afford (*S*)-**1a** as a white solid in 56% yield (160.2 mg, 0.56 mmol). Mp. 44.7-45.5 °C; $[\alpha]^{20}_D$ -44.4 (*c* 1.0, $CHCl_3$); IR (CH_2Cl_2) ν 3464, 3395, 3002, 2938, 2836, 1692, 1594, 1523, 1470, 1368, 1252, 1130, 1085, 1052, 1002, 978, 782, 730, 527 cm^{-1}; ^1H NMR (400 MHz, $CDCl_3$, TMS): δ 1.95 (s, 3H, CH_3), 3.52 (brs, 2H, NH_2), 3.68 (s, 3H, OCH_3), 3.73 (s, 3H, OCH_3), 6.44 (dd, *J* = 8.0, 10.8 Hz, 2H, ArH), 6.78 (d, *J* = 8.4 Hz, 1H, ArH), 7.03 (brs, 1H, NH), 7.18 (t, *J* = 8.0 Hz, 1H, ArH), 7.35 (t, *J* = 8.4 Hz, 1H, ArH), 7.95 (d, *J* = 7.6 Hz, 1H, ArH); ^{13}C NMR (100 MHz, $CDCl_3$): δ 24.7, 55.7, 56.0, 101.2, 106.8, 107.2, 108.6, 113.3, 114.0, 129.3, 130.0, 137.4, 145.9, 157.5, 158.1, 168.3; MS (ESI) *m/e* (%): 287.1 (M^+, 100), 274.3 (M^+-13, 20); HRMS (Micromass LCT) Calcd. for $C_{16}H_{19}N_2O_3$: 287.1396; Found: 287.1393.

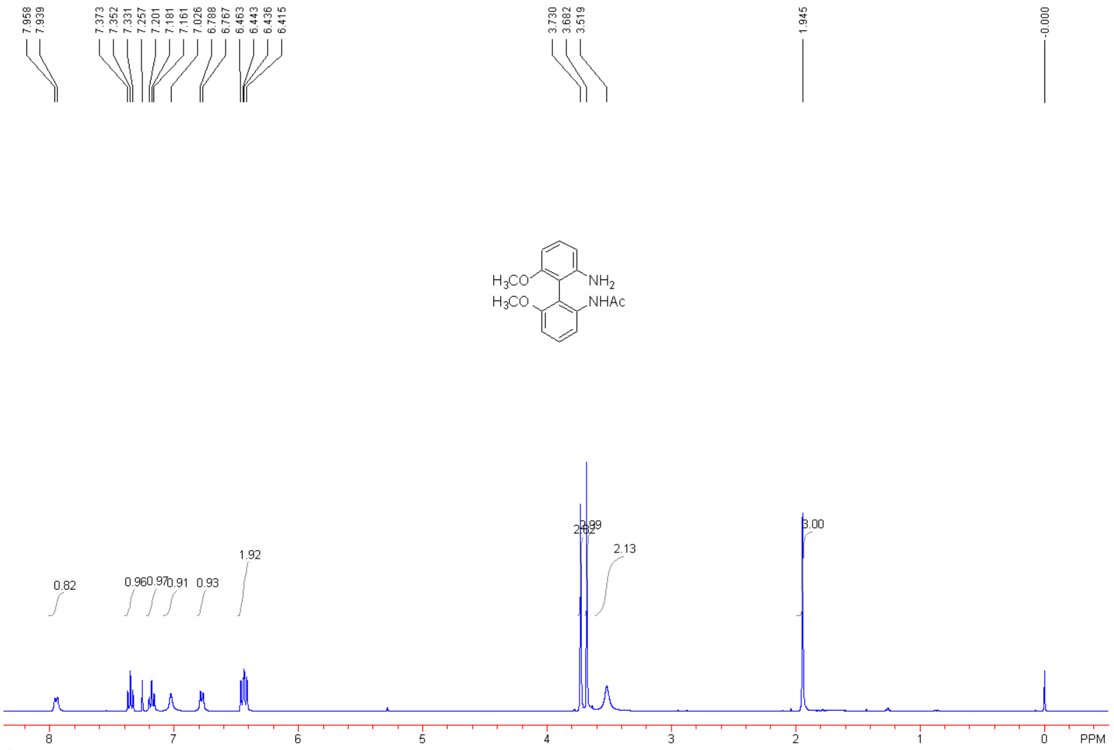

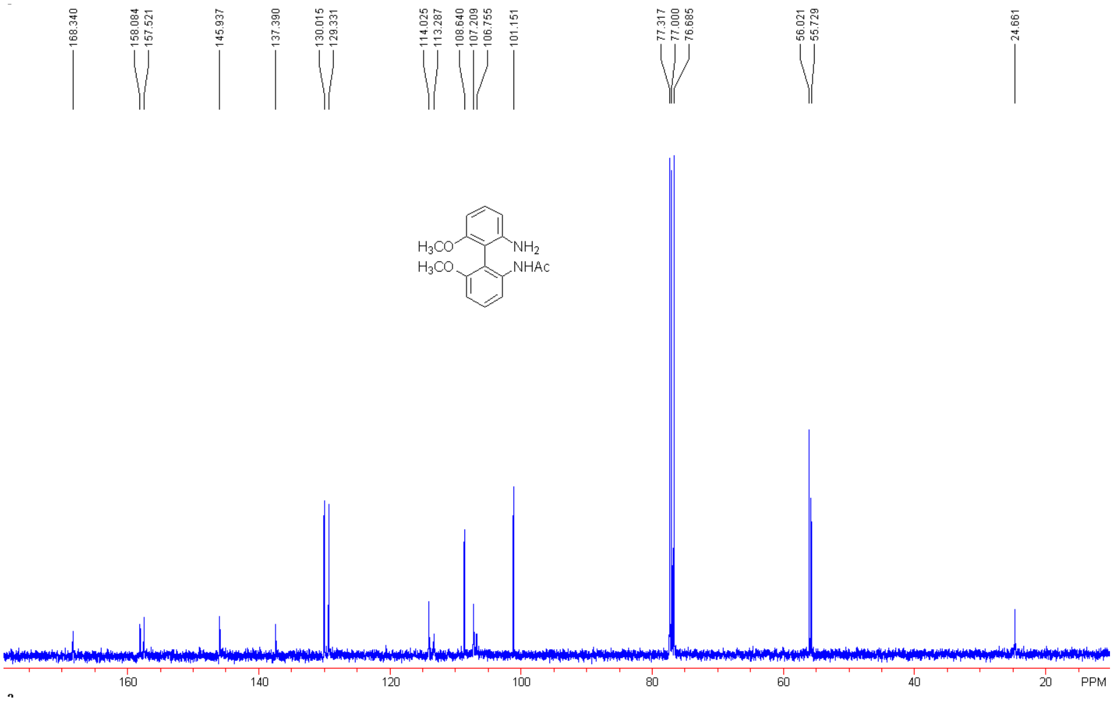

Synthesis of compound (*S*)-2a

Compound (*S*)-**1a** (143.1 mg, 0.50 mmol), 2-bromonitrobenzene (303 mg, 1.5 mmol), $Pd_2(dba)_3$ (12 mg, 0.013 mmol), DPE-phos (20 mg, 0.038 mmol), and Cs_2CO_3 (520 mg, 1.6 mmol) were stirred in anhydrous toluene (4.0 mL) at 80 °C for 48 h. After the reaction mixture was cooled to room temperature, the reaction

was quenched by the addition of 10 mL of H_2O, extracted with EtOAc (2 x 20 mL) and the extracts dried over anhydrous Na_2SO_4. The solvent was removed under reduced pressure and the residue purified by silica gel flash column chromatography (eluent: petroleum ether/ethyl acetate, 20/1) to remove unreacted starting material, and then eluted with petroleum ether/ethyl acetate, 2/1 to give (*S*)-**2a** as a red solid; Yield: 199.5 mg (98%). Mp. 110.8-111.4 °C; $[\alpha]^{20}_{D}$ -139.4 (*c* 1.0, $CHCl_3$); IR (CH_2Cl_2) ν 3335, 2928, 2840, 1682, 1574, 1465, 1252, 1073, 741 cm^{-1}; ^{1}H NMR (400 MHz, $CDCl_3$, TMS): δ 1.74 (s, 0.7H, CH_3), 1.93 (s, 2.3H, CH_3), 3.79 (s, 2.0H, OCH_3), 3.80 (s, 3.3H, OCH_3), 3.87 (s, 0.7H, OCH_3), 6.69 (t, *J* = 8.0 Hz, 1H, ArH), 6.80 (d, *J* = 8.0 Hz, 1H, ArH), 6.91 (d, *J* = 8.4 Hz, 2H, ArH), 7.12 (brs, 0.5H, NH), 7.19 (t, *J* = 8.0 Hz, 1.5H, ArH and NH), 7.28-7.36 (m, 2H, ArH), 7.44 (t, *J* = 7.6 Hz, 1H, ArH), 7.75 (d, *J* = 8.0 Hz, 1H, ArH), 8.07 (dd, *J* = 1.2, 8.8 Hz, 1H, ArH), 9.31 (s, 0.7H, NH), 9.39 (s, 0.3H, NH); ^{13}C NMR (100 MHz, $CDCl_3$): δ 24.5, 55.6, 56.1, 106.7, 107.9, 113.4, 114.7, 116.2, 117.0, 117.4, 126.5, 129.7, 129.8, 133.3, 135.3, 136.8, 139.8, 142.4, 156.6, 157.9, 167.8, 171.1; MS (ESI) *m/e* (%): 430.1 (M^+ + Na, 100), 381.3 (M^+-26, 30.30), 362.3 (M^+-45, 29.51), 318.3 (M^+-89, 29.51); HRMS (Micromass LCT) Calcd. for $C_{22}H_{22}N_3O_5$ 408.1559, Found 408.1559.

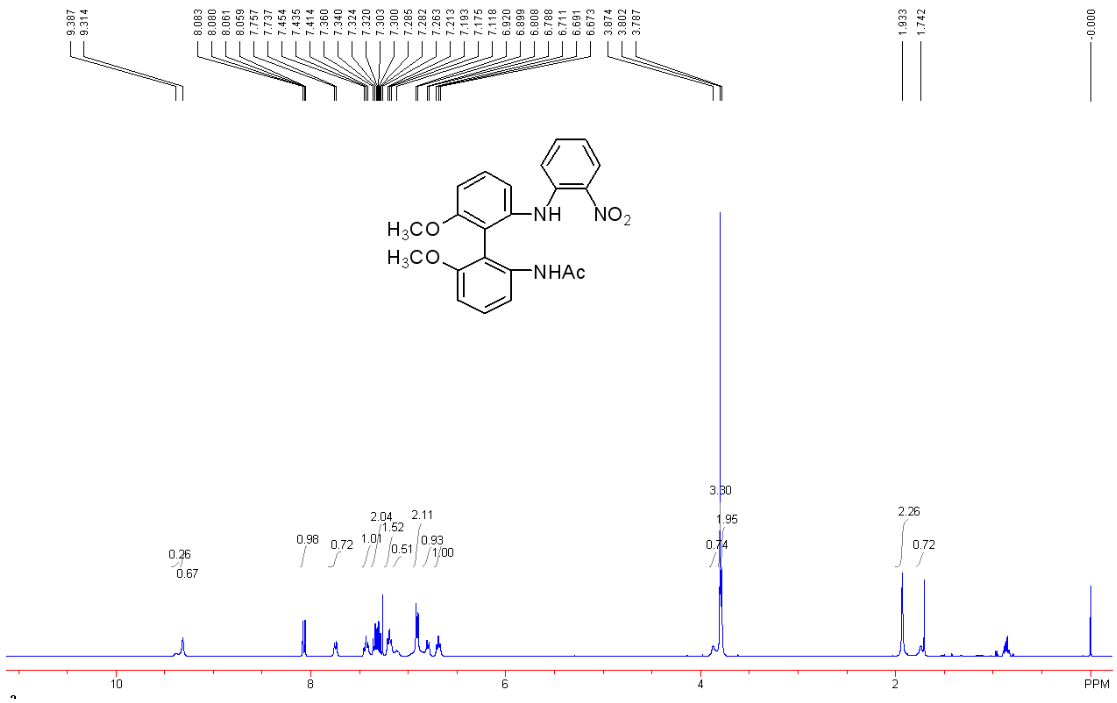

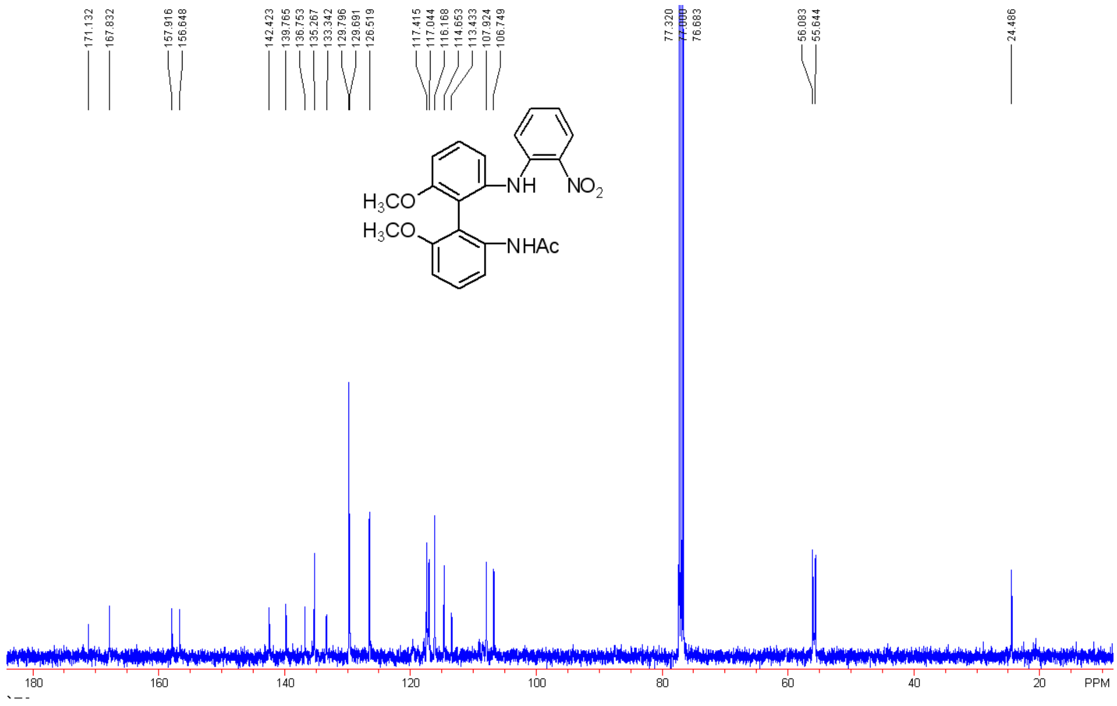

Synthesis of compound (*S*)-3a

A mixture of (*S*)-**2a** (101.8 mg, 0.25 mmol), 10% Pd-C (15 mg) in a mixture of EtOAc and EtOH (15 mL, 1/1) was stirred under a H_2 atmosphere (15 atm) at 60 ^{o}C for 8 h. After cooling to room temperature, Pd-C was removed by filtration. The solvent was evaporated under reduced pressure and the residue purified by silica gel flash column chromatography (eluent: petroleum ether /ethyl acetate, 2/1 - 1/1) to give (*S*)-**3a** as a white solid; Yield: 92.4 mg (98%). Mp. 86.9-87.5 ^{o}C; $[\alpha]^{20}_{D}$ -15.0 (c 0.2, $CHCl_3$); IR (CH_2Cl_2) ν 3396, 2936, 2836, 1689, 1589, 1504, 1468, 1435, 1369, 1254, 1081, 1002, 978, 779, 734, 701 cm^{-1}; ^{1}H NMR (400 MHz, $CDCl_3$, TMS): δ 1.96 (s, 3H, CH_3), 3.55 (brs, 2H, NH_2), 3.71 (s, 3H, OCH_3), 3.73 (s, 3H, OCH_3), 4.88 (brs, 1H, NH), 6.31 (d, *J* = 8.0 Hz, 1H, ArH), 6.47 (d, *J* = 8.4 Hz, 1H, ArH), 6.66-6.71 (m, 2H, ArH), 6.80 (d, *J* = 8.4 Hz, 1H, ArH), 6.99 (t, *J* = 7.2 Hz, 2H, ArH), 7.13-7.24 (m, 2H, ArH). 7.36 (t, *J* = 8.4 Hz, 1H, ArH), 7.92 (brs, 1H, NH); ^{13}C NMR (100 MHz, $CDCl_3$): δ 24.5, 55.7, 55.8, 101.4, 106.7, 107.2, 107.4, 113.4, 114.6, 115.8, 118.5, 126.5, 127.0, 127.1, 129.5, 130.0, 137.8, 143.0, 145.5, 157.2, 157.6, 168.4; MS (ESI) m/e (%): 378.2 (M^{+}+1, 100), 287.1 (M^{+}-103, 18.63); HRMS (Micromass LCT) Calcd. for $C_{22}H_{24}N_3O_3$: 378.1818; Found: 378.1805.

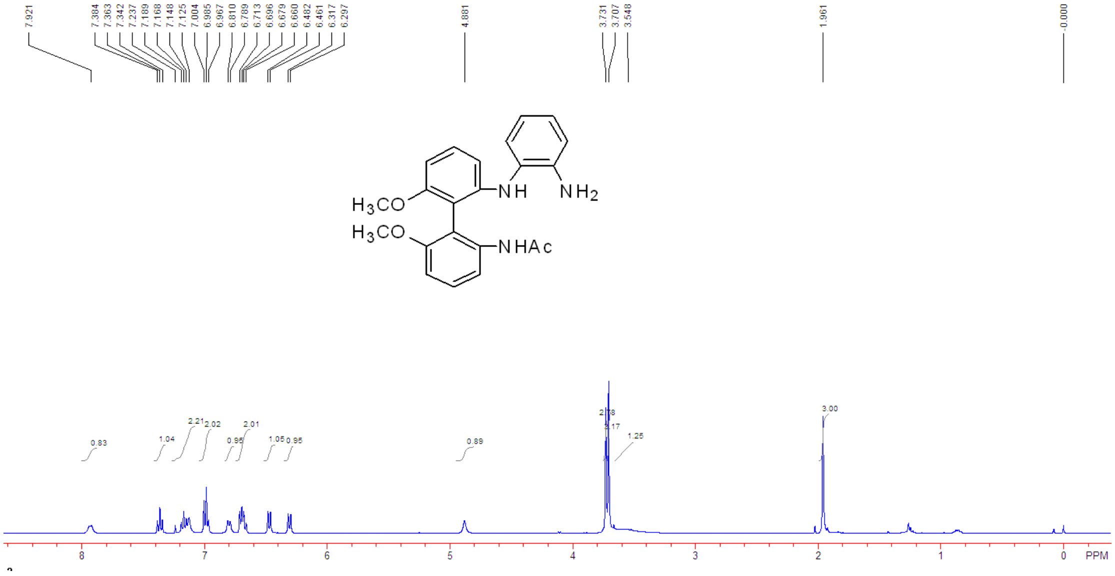

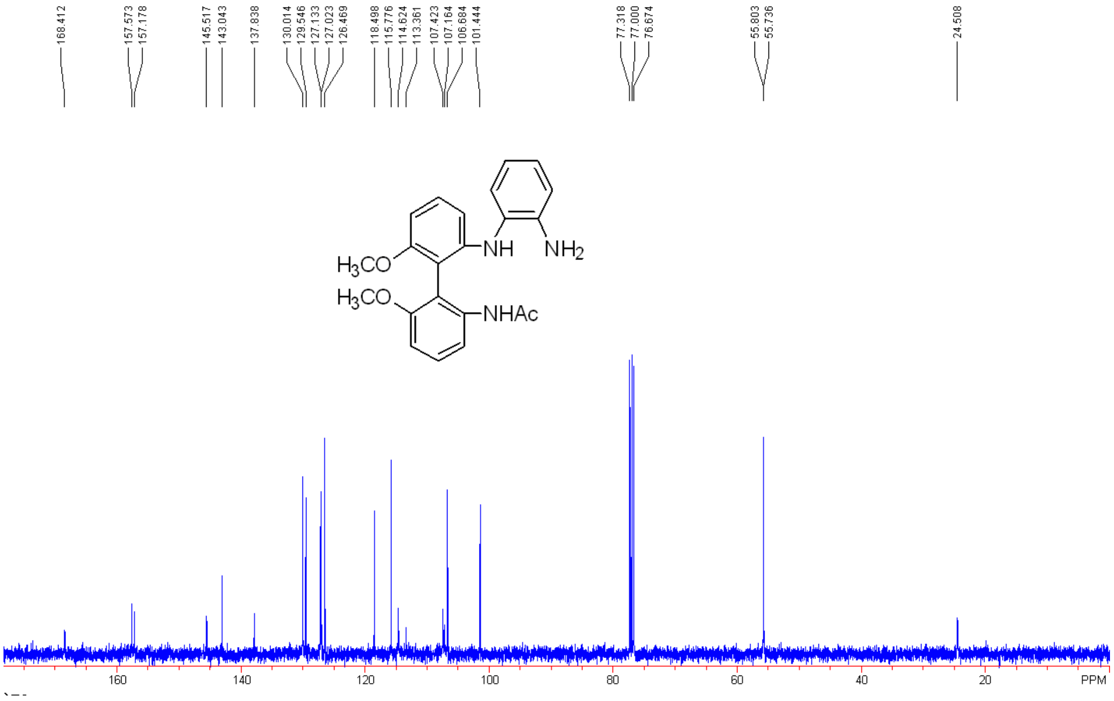

Synthesis of compound (*S*)-4a

Compound (*S*)-**3a** (188.6 mg, 0.50 mmol) and triethyl orthoformate [$HC(OC_2H_5)_3$] (5.0 mL) containing a small amount of TsOH were heated at 100 °C for 5 h. After excess triethyl orthoformate was removed under reduced pressure, the residue was purified by silica gel flash column chromatography (eluent: petroleum ether/ethyl acetate, 2/3) to give (*S*)-**4a** as a white solid; Yield: 170.4 mg (83%). Mp.: 84.8-85.7 °C; $[\alpha]^{20}_{D}$ -63.5 (*c* 0.8, $CHCl_3$); IR (CH_2Cl_2) ν 3243, 2937, 2837, 1681, 1592,1467, 1432, 1259, 1177, 1085, 1009, 892, 778,

743, 637, 560 cm^{-1}; ^{1}H NMR (400 MHz, $CDCl_3$, TMS): δ 1.59 (s, 0.5H, CH_3), 1.91 (s, 2.5H, CH_3), 3.10 (s, 2.5H, OCH_3), 3.41 (s, 0.5H, OCH_3), 3.84 (s, 3H, OCH_3), 6.43 (s, 0.8H, CH), 6.64 (s, 0.2H, CH), 7.16-7.33 (m, 7H, ArH), 7.54-7.70 (m, 4H, ArH and NH); ^{13}C NMR (100 MHz, $CDCl_3$): δ 24.2, 55.0, 56.3, 107.0, 110.0, 111.3, 113.9, 115.7, 119.3, 120.0, 122.2, 123.1, 129.7, 130.2, 134.2, 136.9, 137.2, 142.7, 143.0, 156.6, 158.0, 168.1, 171.1; MS (ESI) *m/e* (%): 388.2 (M^{+}+1, 100), 274.3 (M^{+}-113, 12.50) HRMS (Micromass GCT) Calcd. for $C_{23}H_{21}N_3O_3$: 387.1583; Found: $C_{23}H_{22}N_3O_3$: 388.1661.

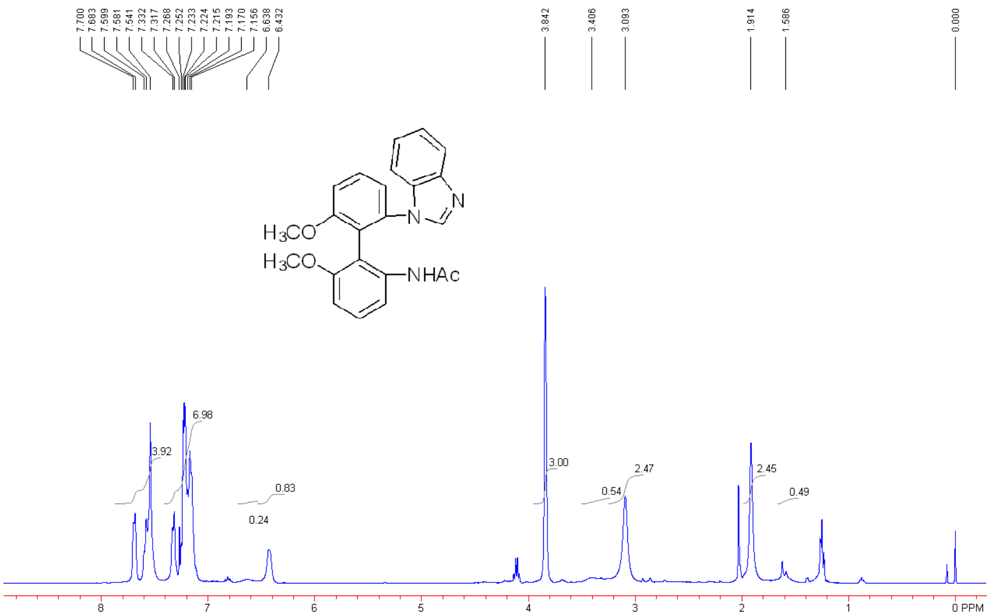

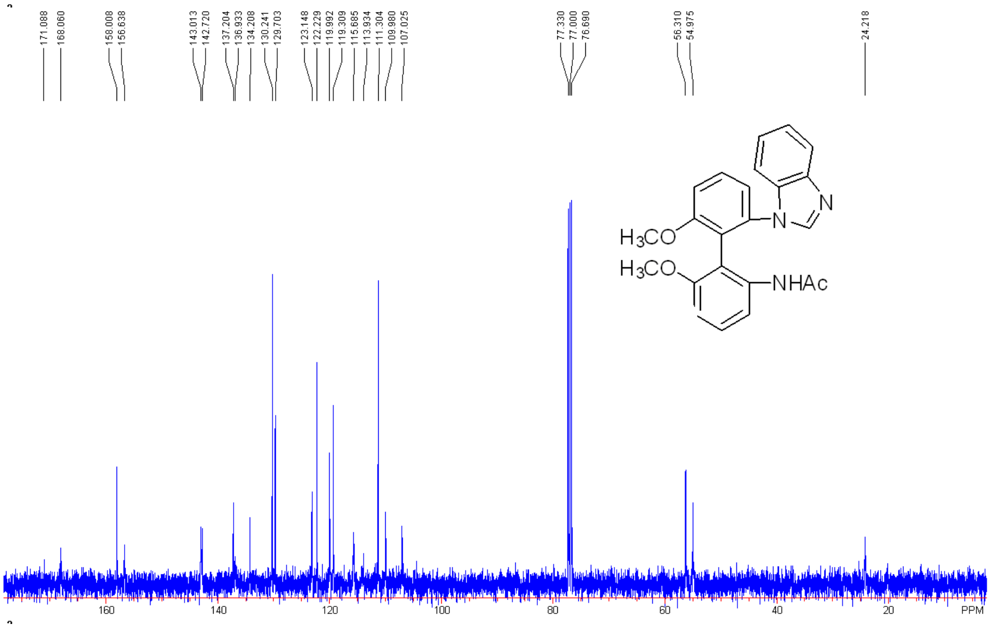

Synthesis of compound (*S*)-5a

Compound (*S*)-**4a** (193.6 mg, 0.50 mmol) and CH_3I (0.60 mL, 10 mmol) in CH_3CN (4.0 mL) were stirred under reflux for 5 h. After cooling to room temperature, the volatiles were removed under reduced pressure and the obtained solid compound (*S*)-**5a** (264.6 mg, 100%) used in the next reaction without further purification. MS (ESI) m/e: 402.2 (M^+-I, 100).

Synthesis of NHC-Pd(II) complex 7

Compound **5a** (105.8 mg, 0.2 mmol), [PdCl(η^3-allyl)]$_2$ (109.1 mg, 0.3 mmol) and *t*-BuOK (56 mg, 0.5 mmol) were refluxed in THF (10 mL) for 8 h. The volatiles were then removed under reduced pressure and the residue was purified by silica gel flash column chromatography (eluent: petroleum ether/ethyl acetate, 2/1-0/1) to give **7** as a mixture of two isomers (117.0 mg, 70%). A single crystal grown from isomeric complex **7** in a saturated solution of CH_2Cl_2/pentane (1/3) was suitable for X-ray crystal structure analysis. (*S*)-**7**, light yellow solid; Mp: 124.6-125.3 °C; $[\alpha]^{20}_D$ +13.0 (*c* 0.25, $CHCl_3$); IR (CH_2Cl_2) ν 3303, 3037, 2933, 2838, 1688, 1688, 1594, 1520, 1464, 1378, 1256, 1125, 1090, 1062, 1004, 976, 939, 733, 560, 530 cm^{-1}; ^1H NMR (400 MHz, $CDCl_3$, TMS): δ [2.07 (s, CH_3), 2.14 (s, CH_3), 1:1.2, 3H], [2.53 (d, *J* = 12.0 Hz, CH_2), 2.71 (d, *J* = 13.2 Hz, CH_2), 1:1.2, 1H], [2.84 (s, OCH_3), 2.88 (s, OCH_3), 1:1.2, 3H], [3.07 (d, *J* = 13.2. Hz, CH_2), 3.65 (d, *J* = 6.8 Hz, CH_2), 1.2:1, 1H], [3.76 (s, OCH_3), 3.79 (s, OCH_3), 1:1.2, 3H], [3.81 (s, CH_3), 3.92 (s, CH_3), 1:1.2, 3H], 4.23 (brs, 1H, CH_2), [4.86-4.95 (m, CH), 5.19-5.29 (m, CH), 1:1.2, 1H], [6.35 (d, *J* = 8.4 Hz, CH_2), 6.39 (d, *J* = 8.0. Hz, CH_2), 1:1.2, 1H], 7.09-7.23 (m, 3H, ArH), 7.29-7.36 (m, 4H, ArH), 7.43-7.69 (m, 3H, ArH), [8.10 (s, NH), 8.18 (s, NH), 1:1.2, 1H]; MS (ESI) *m/z* (%): 675 (M^+, 60.07), 402 (M^+-273, 100), 274 (M^+-401, 28.80); Anal. Calcd. for $C_{27}H_{28}IN_3O_3Pd$ requires: C, 47.98; H, 4.18; N, 6.22%. Found: $C_{27}H_{28}IN_3O_3Pd$, C 47.78, H 4.68, N 5.78%.

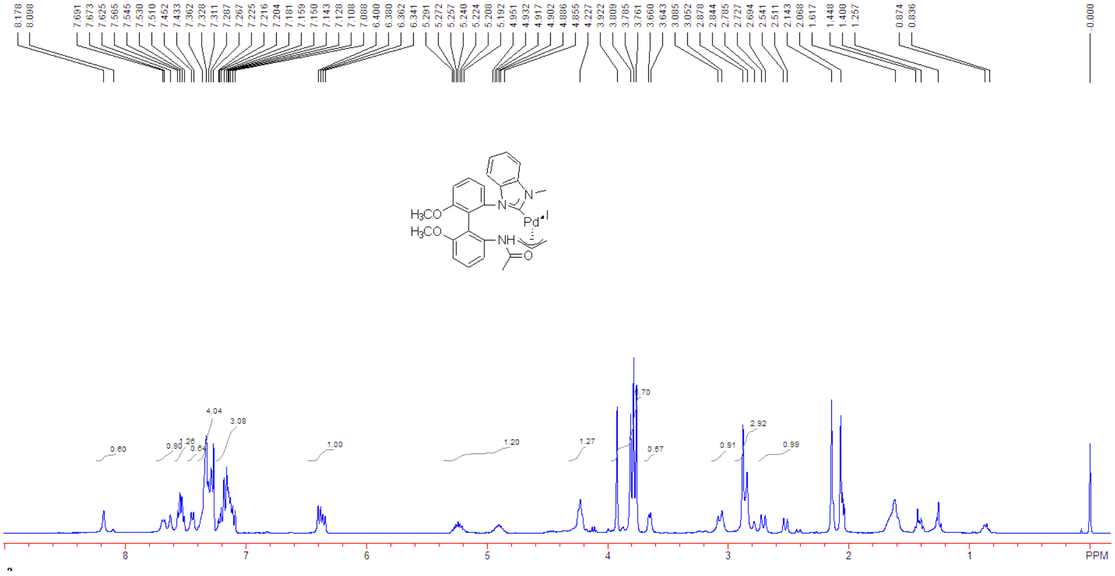

Synthesis of NHC-Au(I) complex (*S*)-6a

Compound (*S*)-**5a** (105.8 mg, 0.2 mmol), AuCl·S(Me)$_2$ (58.8 mg, 0.2 mmol), KI (49.8 mg, 0.3 mmol) and ^{t}BuOK (56 mg, 0.5 mmol) were refluxed in THF (10 mL) for 8 h. The volatiles were then removed under reduced pressure and the residue was purified by silica gel flash column chromatography (eluent: petroleum ether/ethyl acetate, 2/1-0/1) to give **8** as white solid (94 mg, 65%). A single crystal grown from racemic complex **6a** in a saturated solution of CH_2Cl_2/pentane (1/3) was suitable for X-ray crystal structureanalysis. (*S*)-**6a**: Mp: 184.3-129.6 ^{o}C; $[\alpha]^{20}_{D}$ +5.0 (*c* 0.25, $CHCl_3$); IR (CH_2Cl_2) ν 3407, 2929, 2835, 1697, 1591, 1468, 1438, 1286, 1083, 1002, 852, 779, 747, 657 cm^{-1}; ^{1}H NMR (400 MHz, $CDCl_3$, TMS): δ 2.19 (s, CH_3, 3H), 3.21 (s, OCH_3, 3H), 3.81 (s, OCH_3, 3H), 3.97 (s, CH_3, 3H), 6.36 (d, *J* = 8.4 Hz, Ar, 1H), 7.11-7.25(m, Ar and NH, 5H), 7.32-7.37 (m, Ar, 4H), 7.47 (d, *J* = 8.0 Hz, Ar, 1H), 7.62 (t, *J* = 8.0 Hz, Ar, 1H); MS (ESI) *m/z* (%): 551 (M^+, 10.05), 598 (M^+-127, 100), 612 (M^+-113, 22.10); Anal. Calcd. for $C_{24}H_{23}IN_3O_3Au$ requires: C, 39.74; H, 3.20; N, 5.79%. Found: $C_{24}H_{23}IN_3O_3Au$ C 40.64, H 3.08, N 5.72%.

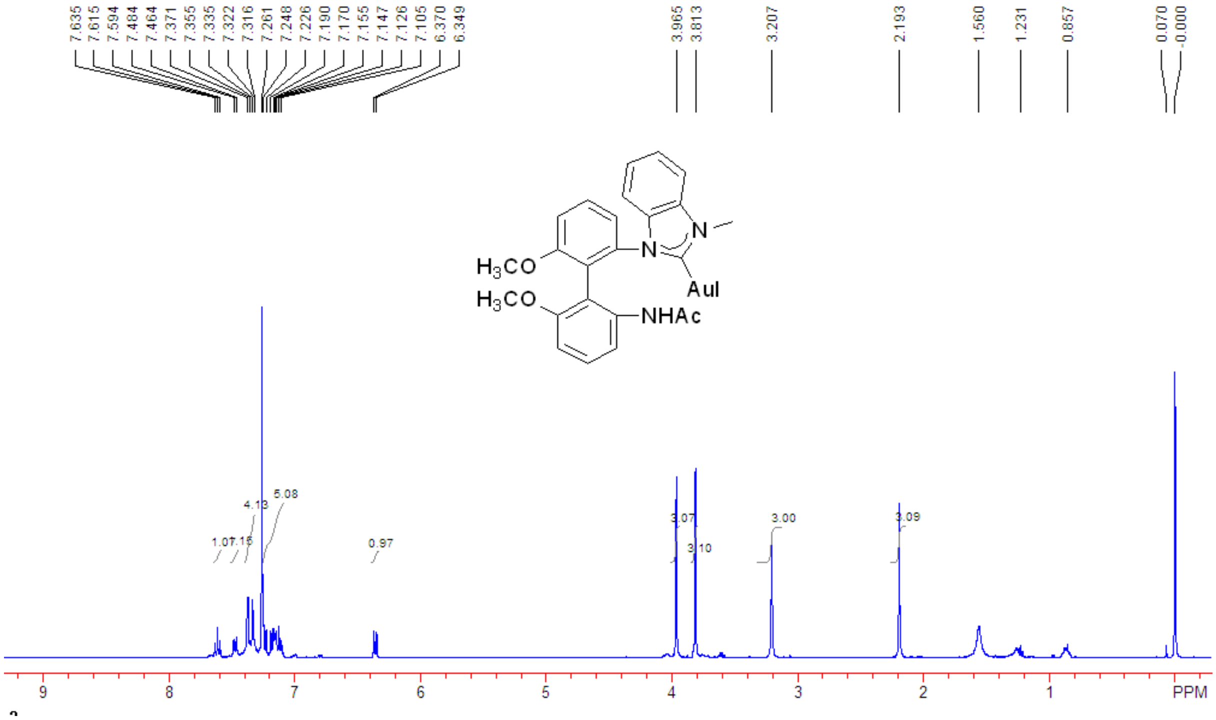

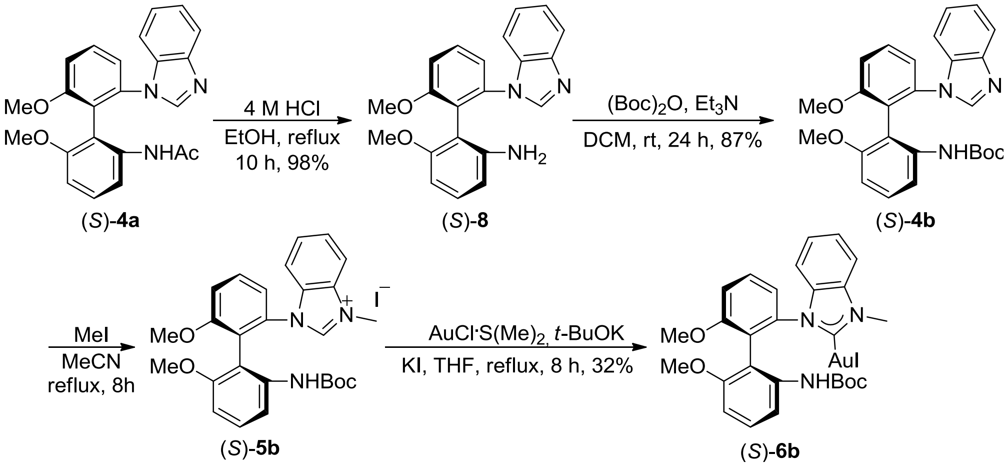

Synthesis of (*S*)-8

4.0 M HCl 10 mL was added to a solution of (*S*)-**4a** (967.9 mg, 25.0 mmol) in 50 mL of EtOH, and then heated under reflux for 10 h. The resulting solution was cooled to room temperature and 2.0 N aqueous NaOH solution added to adjust the pH to ≈ 7. The reaction mixture was extracted by CH_2Cl_2 (3 x 20 mL) and the combined organic phases were washed with saturated brine and dried over $MgSO_4$. The solvent was removed under reduced pressure and the crude product purified by flash chromatography (ethyl acetate/hexane = 2/1)

to afford (*S*)-**8** as white solid in 98% yield (845.6 mg, 24.5 mmol). Mp. 81.7-82.5 °C; $[\alpha]^{20}_{D}$ -34.0 (*c* 0.5, $CHCl_3$); IR (CH_2Cl_2) ν 2930, 2854, 1615, 1589, 1489, 1469, 1436, 1261, 1117, 1087, 838, 800, 745 cm^{-1}; ^{1}H NMR (400 MHz, $CDCl_3$, TMS): δ 3.14 (s, 3H, OCH_3), 3.54 (brs, 2H, NH_2), 3.86 (s, 3H, OCH_3), 6.04 (d, *J* = 8.0 Hz, 1H, ArH), 6.29 (d, *J* = 8.0 Hz, 1H, ArH), 6.96 (t, *J* = 8.0 Hz, 1H, ArH), 7.14 (d, *J* = 8.4 Hz, 1H, ArH), 7.19-7.22 (m, 3H, Ar), 7.39-7.41 (m, 1H, Ar), 7.54 (t, *J* = 8.0 Hz, 1H, ArH), 7.63 (s, 1H, ArH), 7.69-7.71 (m, 1H, ArH); ^{13}C NMR (100 MHz, $CDCl_3$): δ 55.0, 56.3, 100.8, 108.0, 108.6, 110.3, 111.3, 119.2, 119.9, 121.3, 121.9, 122.9, 129.6, 129.8, 134.4, 137.1, 143.0, 143.3, 145.9, 157.3, 158.5; MS (ESI) *m/e* (%): 346.2 (M + H^+, 100); HRMS (Micromass LCT) Calcd. for $C_{21}H_{20}N_3O_2$: 346.1556; Found: $C_{21}H_{20}N_3O_2$, 346.1554.

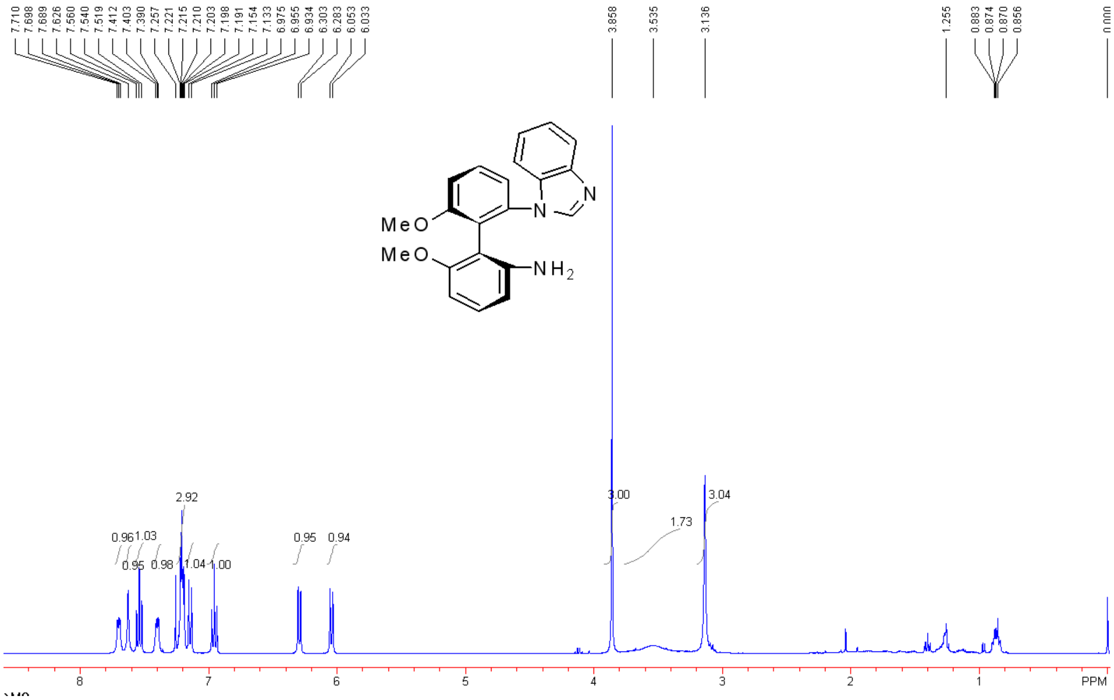

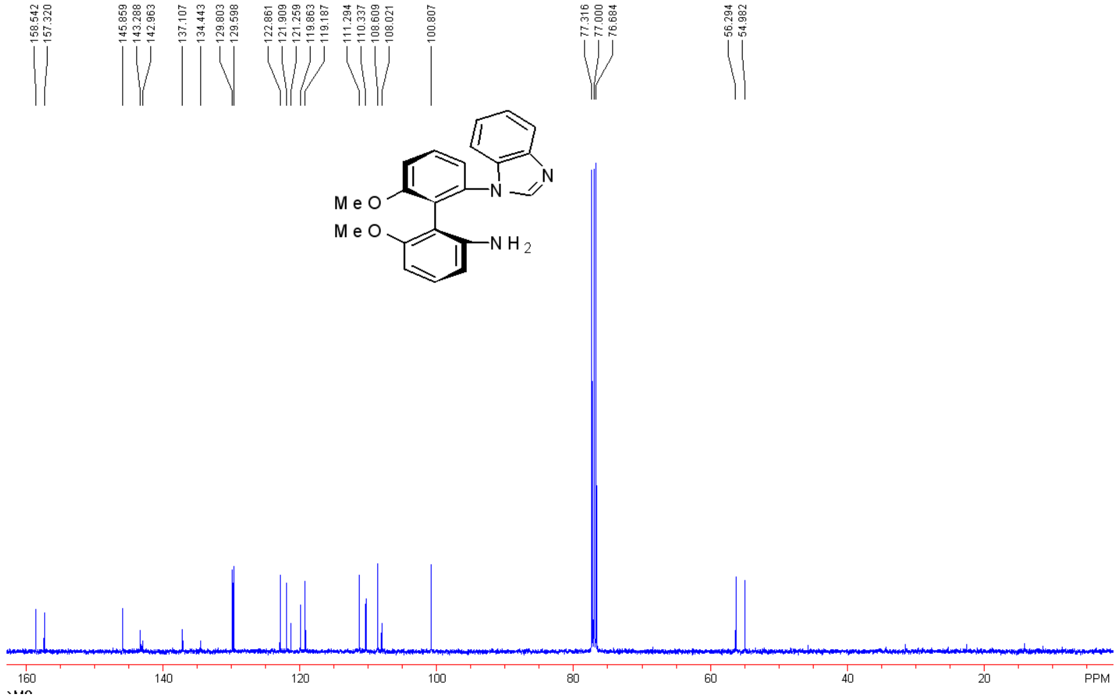

Synthesis of (*S*)-4b

Compound (*S*)-**8** (172.6 mg, 0.50 mmol), $(Boc)_2O$ (218.1 mg, 1.0 mmol) and Et_3N (101.1 mg, 1.0 mmol) were dissolved in 10 mL of anhydrous CH_2Cl_2. The reaction was stirred at room temperature for 24 h, then the solvent was removed under reduced pressure and the crude product purified by flash chromatography (ethyl acetate/hexane = 4/1) to afford (*S*)-**4b** as white solid in 87% yield (193.7 mg, 0.435 mmol). Mp. 99.6-101.0 ^oC; $[\alpha]^{20}_D$ -42.5 (*c* 0.5, $CHCl_3$); IR (CH_2Cl_2) ν 3430, 3004, 2981, 2934, 1733, 1593, 1471, 1431, 1367, 1294, 1241, 1160, 1048, 889, 763 cm^{-1}; ^1H NMR (400 MHz, $CDCl_3$, TMS): δ 1.41 (s, 9H, CH_3), 2.44 (brs, 0.3H, OCH_3), 3.14 (brs, 2.7H, OCH_3), 3.82 (s, 3H, OCH_3), 6.25 (brs, 1H, NH), 6.35 (d, J = 8.4 Hz, 1H, Ar), 7.10-7.23 (m, 5H, ArH), 7.32-7.35 (m, 1H, ArH), 7.45 (d, J = 7.6 Hz , 1H, ArH), 7.54-7.59 (m, 2H, ArH), 7.68-7.70 (m, 1H, ArH); ^{13}C NMR (100 MHz, $CDCl_3$): δ 28.1, 54.9, 56.1, 80.3, 105.6, 110.0, 111.3, 119.1, 119.7, 119.8, 122.0, 123.0, 129.5, 130.1, 134.2, 136.9, 137.5, 142.8, 142.9, 152.6, 156.5, 158.1, 171.0; MS (ESI) *m/e* (%): 446.2 ($M + H^+$, 100); HRMS (Micromass LCT) Calcd. for $C_{26}H_{28}N_3O_4$: 446.2080; Found: $C_{26}H_{28}N_3O_4$, 446.2081.

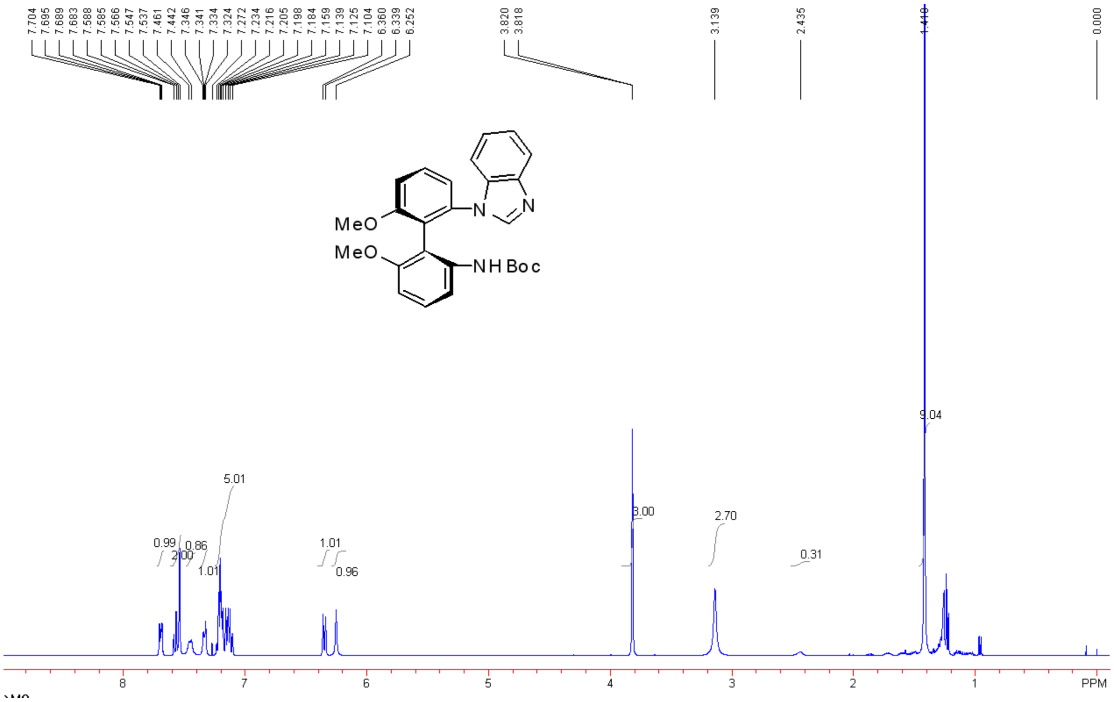

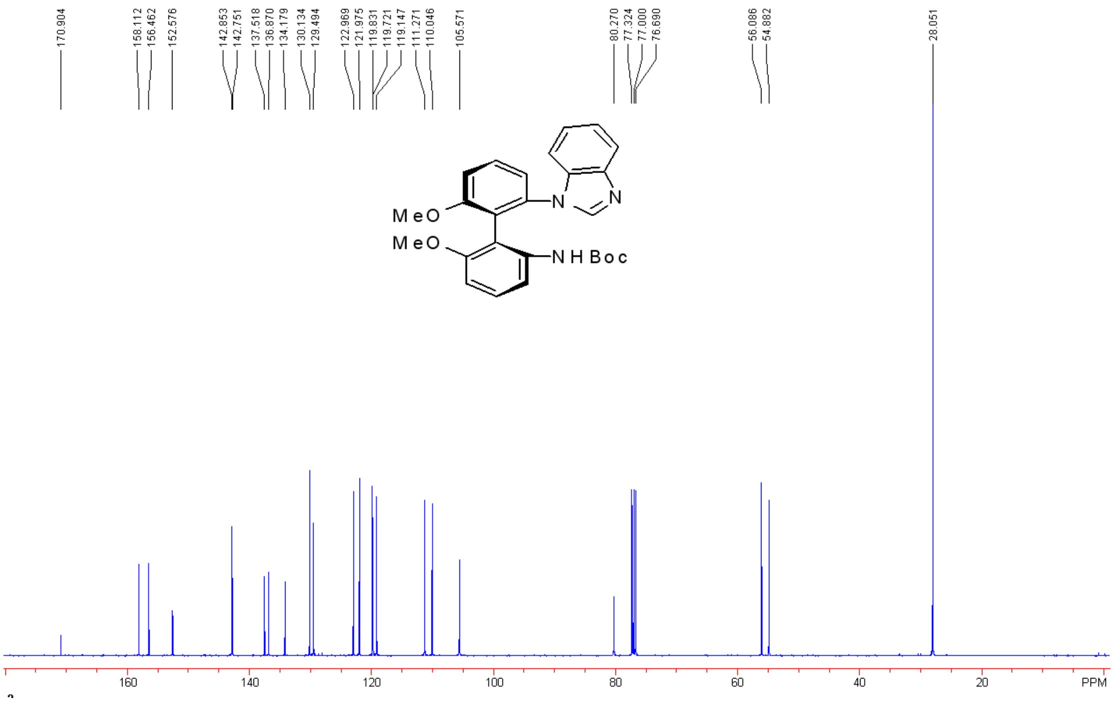

Synthesis of (*S*)-5b

Compound (*S*)-**4b** (222.6 mg, 0.50 mmol) and CH_3I (0.60 mL, 10 mmol) in CH_3CN (4.0 mL) were stirred under reflux for 8 h. After cooling to room temperature, the volatiles were removed under reduced pressure and the solid obtained (*S*)-**5b** used for the next reaction without further purification. MS (ESI) *m/e:* 460.2 (M^+-I, 100).

Synthesis of NHC-Au(I) complex (*S*)-6b

Compound (S)-**5b** (117.4 mg, 0.2 mmol), AuCl·S(Me)$_2$ (58.8 mg, 0.2 mmol), KI (49.8 mg, 0.3 mmol) and ^{t}BuOK (56 mg, 0.5 mmol) were refluxed in THF (10 mL) for 8 h. The volatiles were then removed under reduced pressure and the residue was purified by silica gel flash column chromatography (eluent: petroleum ether/ethyl acetate, 2/1-0/1) to give (S)-**6b** as white solid (50.0 mg, 32%). Mp. 233.3-234.7 °C; $[\alpha]^{20}_D$ -26.8 (c 0.5, CHCl$_3$); IR (CH$_2$Cl$_2$) ν 3006, 1592, 1469, 1431, 1276, 1259, 1157, 1048, 751 cm^{-1}; ^{1}H NMR (400 MHz, CDCl$_3$, TMS): δ 1.55 (s, 9H, CH$_3$), 3.33 (s, 3H, OCH$_3$), 3.83 (s, 3H, OCH$_3$), 3.96 (s, 3H, CH$_3$), 6.26 (brs, 1H, NH), 6.31 (d, J = 8.0 Hz, 1H, Ar), 7.06 (t, J = 8.4 Hz, 1H, Ar), 7.21-7.28 (m, 4H, Ar), 7.31-7.33 (m, 2H, Ar), 7.41 (d, J = 8.8 Hz, 1H, Ar), 7.60 (t, J = 8.0 Hz, 1H, Ar); ^{13}C NMR (100 MHz, CDCl$_3$): δ 28.6, 34.8, 54.8, 56.2, 80.7, 104.9, 110.7, 112.0, 112.7, 113.1, 114.0, 121.0, 121.2, 123.9, 124.3, 129.3, 130.4, 132.3, 134.1, 137.5, 137.7, 152.8, 156.3, 158.8, 188.2; MS (ESI) m/e (%): 425 (M$^+$ + Na, 100); MS (ESI) m/e (%): 656.2 (M$^+$-I, 100); HRMS (Micromass LCT) Calcd. for $C_{27}H_{29}N_3O_4Au^+$ 656.1824, Found $C_{27}H_{29}N_3O_4Au$ 656.1848.

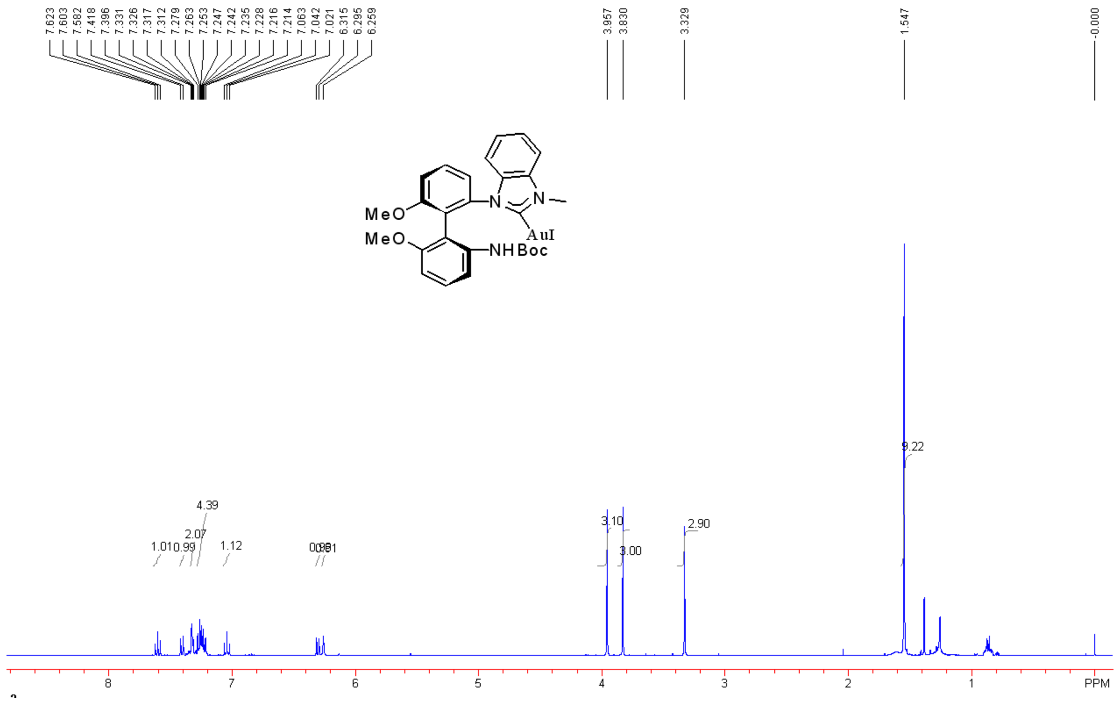

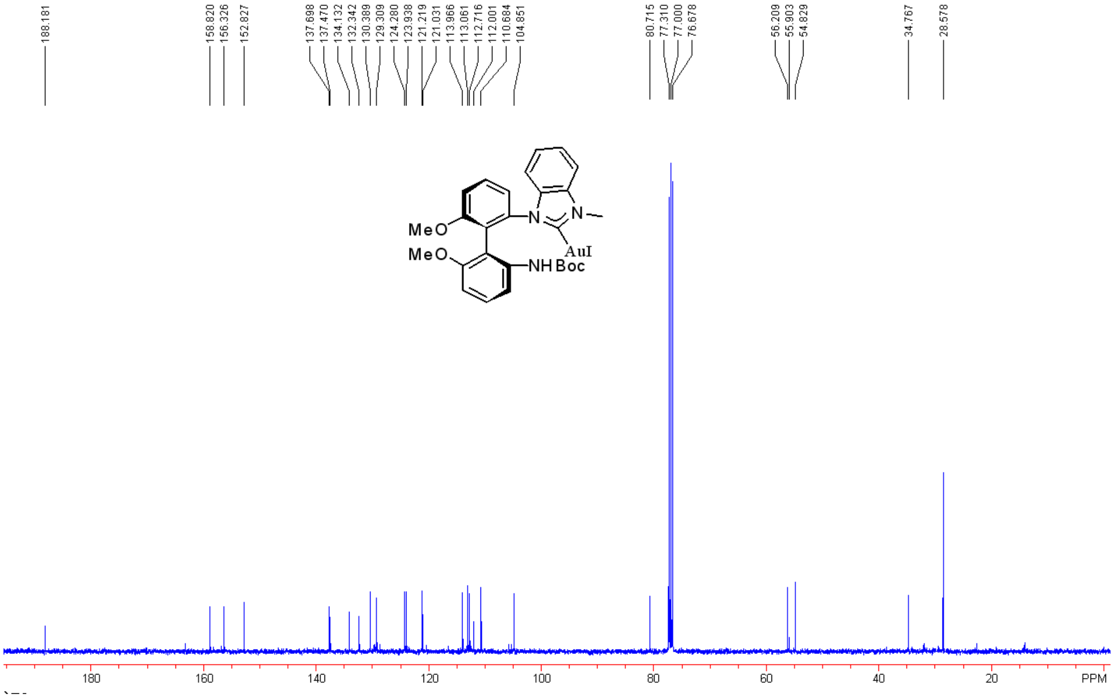

188.181
158.820
156.326
152.827
137.698
137.470
134.132
132.342
130.389
129.309
124.280
123.938
121.219
121.031
113.966
113.061
112.716
112.001
110.684
104.851
80.715
77.310
77.000
76.678
56.209
55.903
54.829
34.767
28.578
MeO
MeO
NHBoc
AuI
180
160
140
120
100
80
60
40
20
PPM

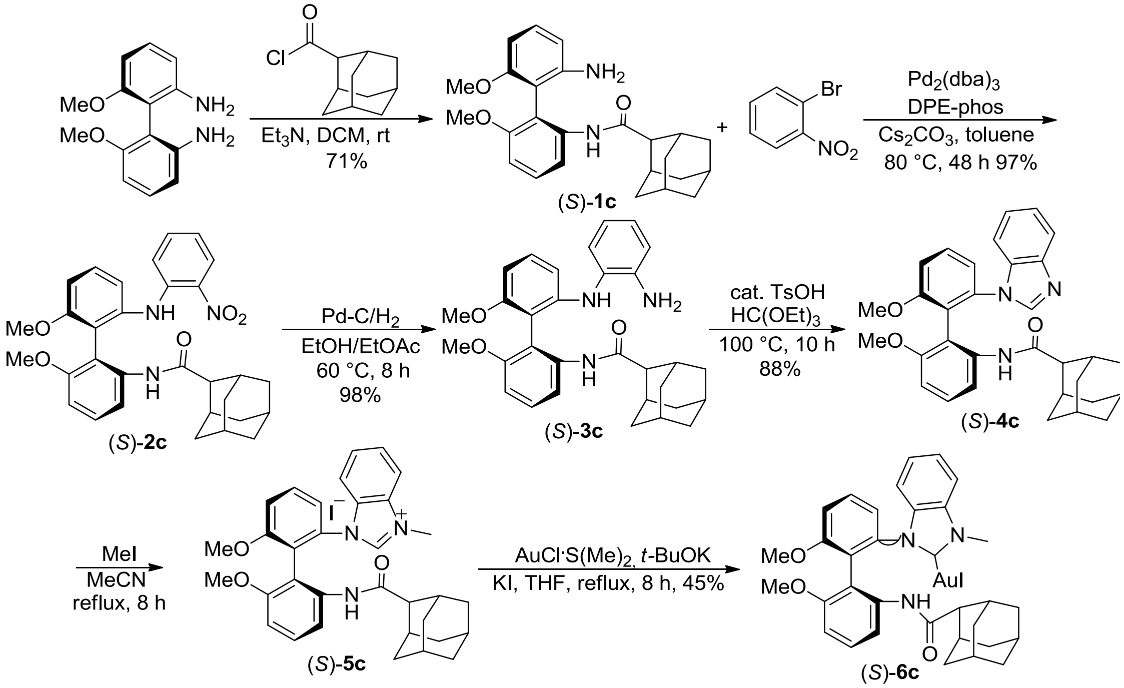

Et3N, DCM, rt
71%
(S)-1c
Pd2(dba)3
DPE-phos
Cs2CO3, toluene
80 °C, 48 h 97%
(S)-2c
Pd-C/H2
EtOH/EtOAc
60 °C, 8 h
98%
(S)-3c
cat. TsOH
HC(OEt)3
100 °C, 10 h
88%
(S)-4c
MeI
MeCN
reflux, 8 h
(S)-5c
AuCl·S(Me)2, t-BuOK
KI, THF, reflux, 8 h, 45%
(S)-6c

Synthesis of (*S*)-1c

Adamantane-2-carbonyl chloride (198.0 mg, 1.0 mmol) in 10 mL of dry CH_2Cl_2 was added to the mixture of 6,6'-dimethoxybiphenyl-2,2'-diamine (244.1 mg, 1.0 mmol) and Et_3N (1.0 mL) in dry CH_2Cl_2 (200 mL) at 0 °C, and the resulting solution stirred for overnight at room temperature. The solvent was removed under reduced pressure and the crude product purified by flash chromatography (ethyl acetate/hexane = 6/1) to afford (*S*)-**1c** as white solid in 71% yield (288.4 mg, 0.71 mmol). Mp. 44.7-45.5 °C; $[\alpha]^{20}_D$ -44.4 (*c* 1.0, $CHCl_3$); IR (CH_2Cl_2) ν 3464, 3395, 3002, 2938, 2836, 1692, 1594, 1523, 1470, 1368, 1252, 1130, 1085, 1052, 1002, 978, 782, 730, 527 cm^{-1}; ^1H NMR (400 MHz, $CDCl_3$, TMS): δ 1.57-1.69 (m, 12H, CH and CH_2), 1.95 (brs, 3H, CH), 2.94 (brs, 2H, NH_2), 3.68 (s, 3H, OCH_3), 3.75 (s, 3H, OCH_3), 6.44 (d, *J* = 8.4 Hz, 1H, ArH), 6.47 (d, *J* = 8.4 Hz, 1H, ArH), 6.77 (d, *J* = 8.0 Hz , 1H, ArH), 7.19 (t, *J* = 8.0 Hz, 1H, ArH), 7.34 (d, *J* = 8.4 Hz, 1H, ArH), 7.37 (brs, 1H, NH), 8.00 (d, *J* = 8.0 Hz, 1H, ArH); ^{13}C NMR (100 MHz, $CDCl_3$): δ 28.0, 36.3, 38.8, 41.5, 55.7, 56.0, 101.0, 106.8, 106.9, 108.6, 113.1, 113.6, 129.4, 130.1, 137.5, 145.9, 157.5, 157.9, 175.9; MS (ESI) *m/e:* 429.2 (M^+ + Na, 100). HRMS (Micromass LCT) Calcd. for $C_{25}H_{30}N_2O_3Na$: 429.2154; Found: $C_{25}H_{30}N_2O_3Na$, 429.2144.

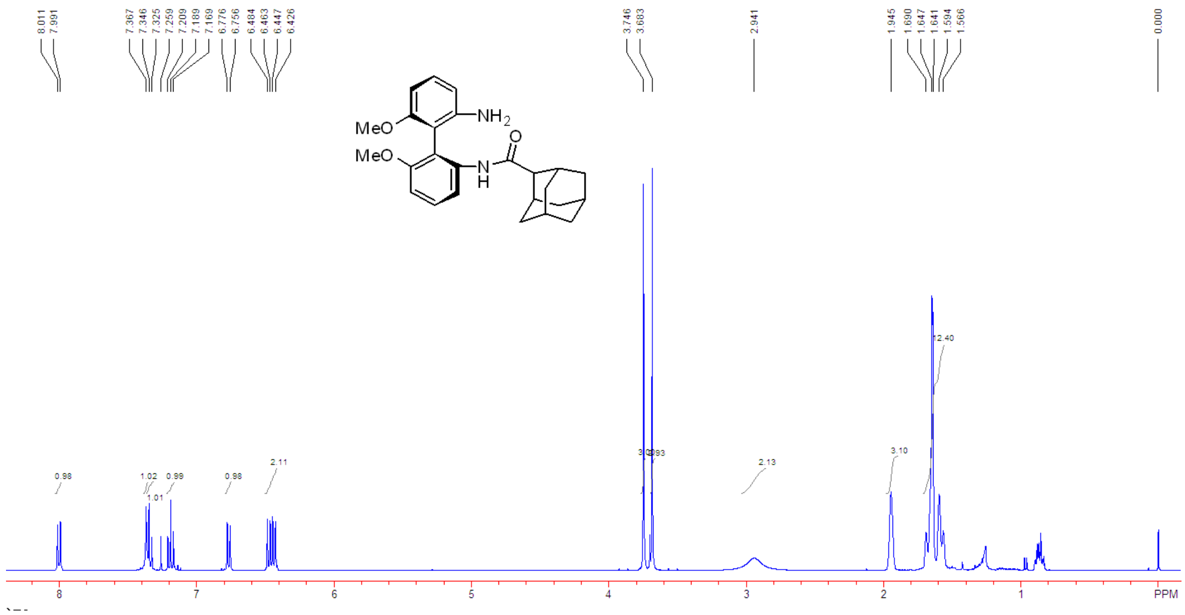

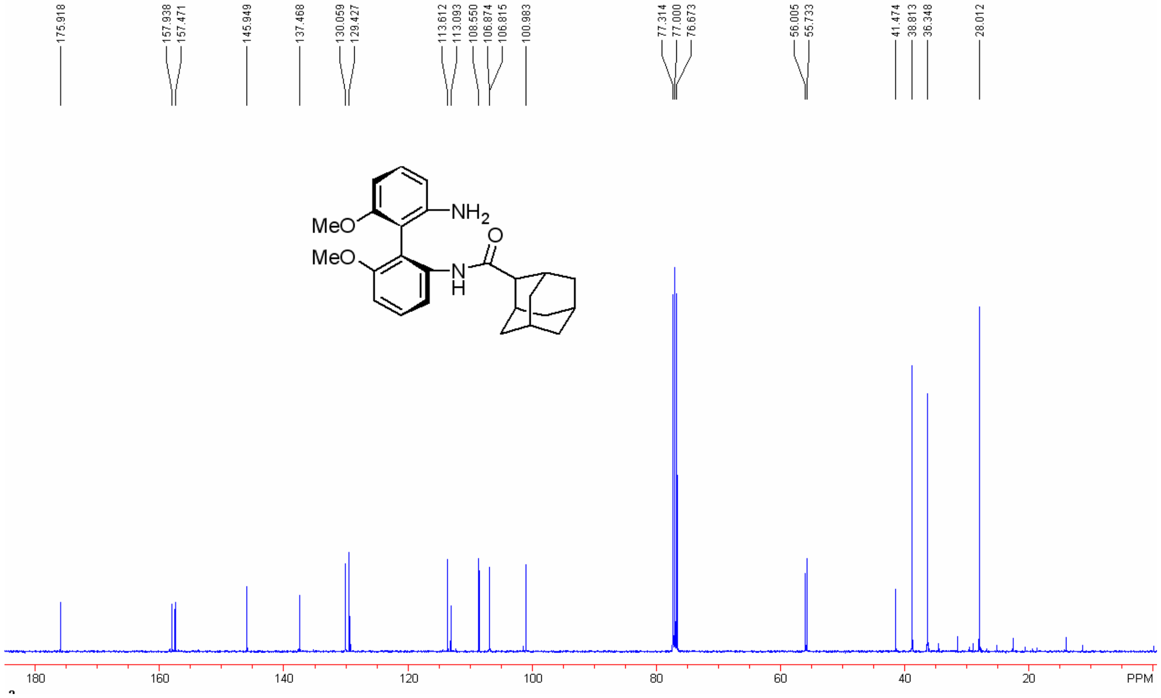

Synthesis of (*S*)-2c

Compound (*S*)-**1c** (203.1 mg, 0.50 mmol), 2-bromonitrobenzene (303 mg, 1.5 mmol), $Pd_2(dba)_3$ (12 mg, 0.013 mmol), DPE-phos (20 mg, 0.038 mmol), and Cs_2CO_3 (520 mg, 1.6 mmol) were stirred in anhydrous toluene (4.0 mL) at 80 °C for 48 h. After the reaction mixture was cooled to room temperature, the reaction was quenched by addition of 10 mL of H_2O. The organic compound was extracted with EtOAc (2 x 20 mL) and dried over anhydrous Na_2SO_4. The solvent was removed under reduced pressure and the residue purified by silica gel flash column chromatography (eluent: petroleum ether/ethyl acetate, 20/1) to remove unreacted starting material, and then eluted with petroleum ether/ethyl acetate, 8/1 to give (*S*)-**2c** as red solid; yield: 255.7 mg (97%). Mp. 110.8-111.4 °C; $[\alpha]^{20}_{D}$ -5.5 (*c* 0.5, $CHCl_3$); IR (CH_2Cl_2) ν 3427, 3339, 2924, 2852, 1686, 1617, 1576, 1466, 1435, 1271, 1074, 785 cm^{-1}; ^1H NMR (400 MHz, $CDCl_3$, TMS): δ 1.59-1.73 (m, 12H, CH and CH_2), 1.98 (brs, 3H, CH), 3.81 (s, 3H, OCH_3), 3.82 (s, 3H, OCH_3), 6.66-6.70 (m, 1H, ArH), 6.78 (d, *J* = 8.4 Hz, 1H, ArH), 6.93 (d, *J* = 8.0 Hz, 1H, ArH), 7.11 (d, *J* = 8.4 Hz, 1H, ArH), 7.19 (d, *J* = 8.0 Hz, 1H, ArH), 7.25-7.29 (m, 2H, ArH), 7.32 (t, *J* = 8.4 Hz, 1H, ArH), 7.46 (t, *J* = 8.4 Hz, 1H, ArH), 7.85 (d, *J* = 8.0 Hz, 1H, ArH), 8.06 (dd, *J* = 0.8, 8.4 Hz, 1H, ArH), 9.31 (s, 1H, NH); ^{13}C NMR (100 MHz, $CDCl_3$): δ 28.0, 36.4, 39.1, 41.4,

55.6, 56.0, 106.3, 107.9, 113.1, 114.0, 116.1, 117.3, 117.4, 117.8, 126.5, 129.78, 129.83, 133.4, 135.2, 136.9, 139.8, 142.5, 156.6, 157.9, 175.3; MS (ESI) *m/e* (%): 550.2 (M^+ + Na, 100); HRMS (Micromass LCT) Calcd. for $C_{31}H_{33}N_3O_5Na$: 550.2318; Found: $C_{31}H_{33}N_3O_5Na$, 550.2313.

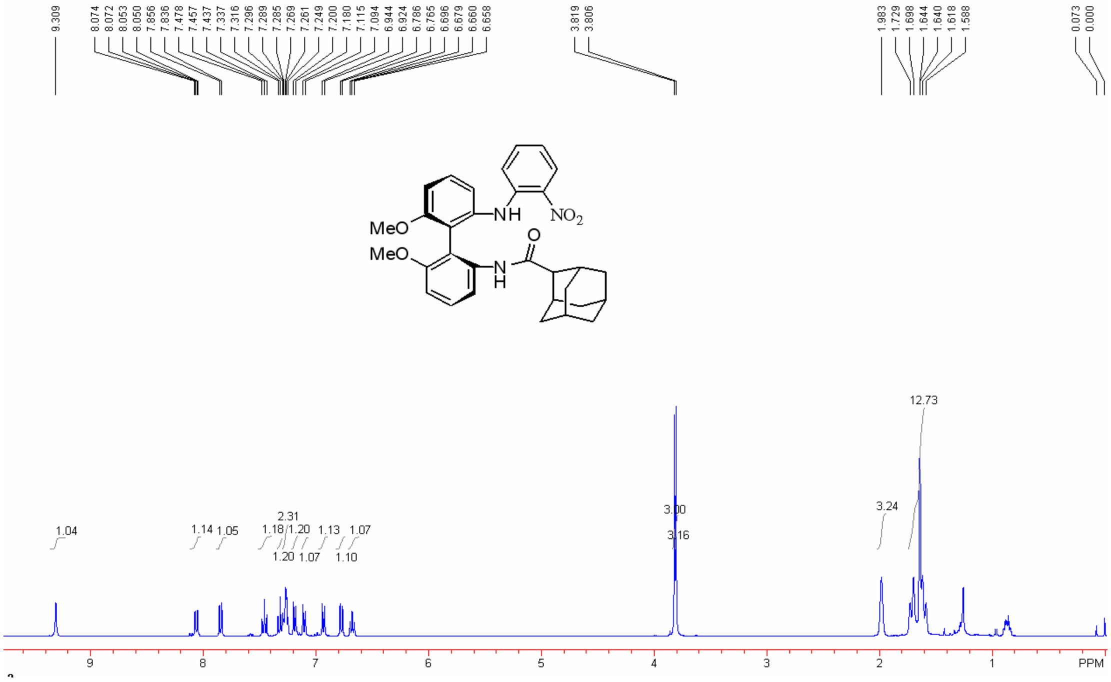

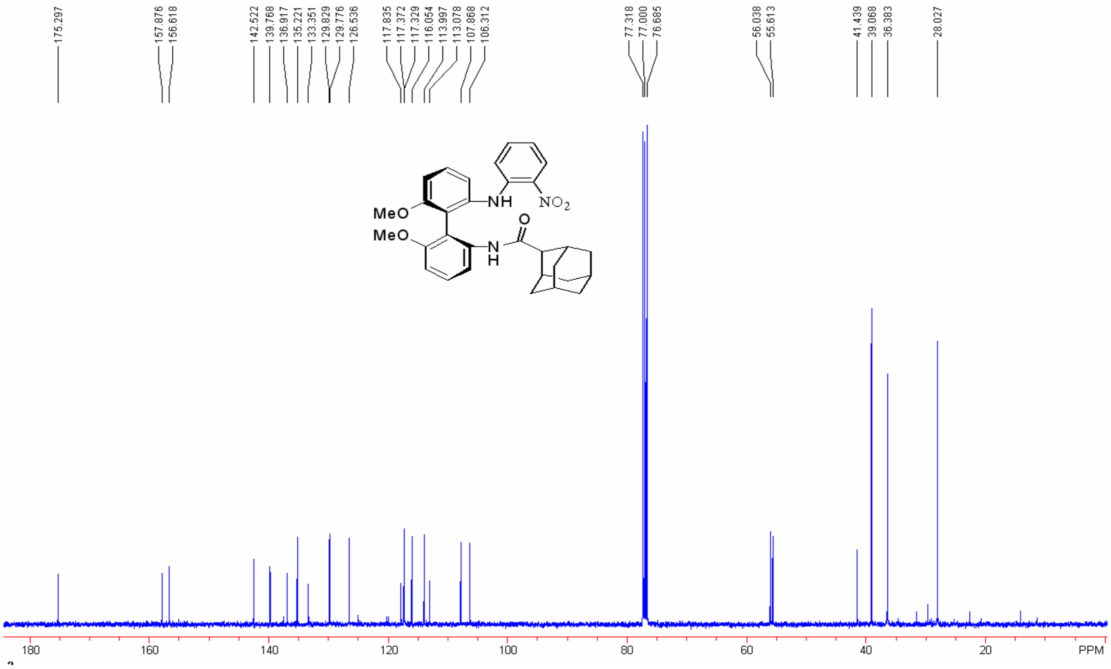

Synthesis of (*S*)-3c

A mixture of (*S*)-**2c** (131.8 mg, 0.25 mmol), 10% Pd-C (15 mg) in a mixture of EtOAc and EtOH (15 mL, 1/1) were stirred under a H_2 atmosphere (15 atm) at 60 °C for 8 h. After cooling to room temperature, Pd-C was removed by filtration. The solvent was evaporated under reduced pressure. The residue was purified by silica gel flash column chromatography (eluent: petroleum ether/ethyl acetate, 2/1 - 1/1) to give (*S*)-**3c** as a white solid; yield: 121.8 mg (98%). $[\alpha]^{20}_{D}$ +19.9 (*c* 0.5, $CHCl_3$); IR (CH_2Cl_2) ν 3500, 2962, 2921, 2851, 1682, 1587, 1503, 1259, 1088, 1018, 798, 743, 703; ^{1}H NMR (400 MHz, $CDCl_3$, TMS): δ 1.57-1.70 (m, 12H, CH and CH_2), 1.96 (brs, 3H, CH), 3.73 (s, 3H, OCH_3), 3.77 (s, 3H, OCH_3), 4.87 (brs, 1H, NH), 6.34 (d, *J* = 8.0 Hz, 1H, ArH), 6.49 (d, *J* = 8.0 Hz, 1H, ArH), 6.67-6.73 (m, 2H, ArH), 6.81 (d, *J* = 8.4 Hz, 1H, ArH), 6.98-7.01 (m, 2H, ArH), 7.18 (t, *J* = 8.0 Hz, 1H, ArH), 7.38 (t, *J* = 8.0 Hz, 1H, ArH), 7.42 (brs, 1H, NH), 7.95 (d, *J* = 8.0 Hz, 1H, ArH); ^{13}C NMR (100 MHz, $CDCl_3$): δ 28.0, 36.4, 38.9, 41.5, 55.8, 55.9, 101.2, 106.7, 107.3, 113.6, 114.6, 115.7, 118.5, 126.5, 127.1, 127.3, 129.7, 130.1, 138.0, 143.1, 145.5, 157.2, 157.5, 176.3; MS (ESI) *m/e* (%): 520.3 (M^+ + Na, 100); HRMS (Micromass LCT) Calcd. for $C_{31}H_{35}N_3O_3Na$ 520.2576, Found $C_{31}H_{35}N_3O_3Na$ 520.2565.

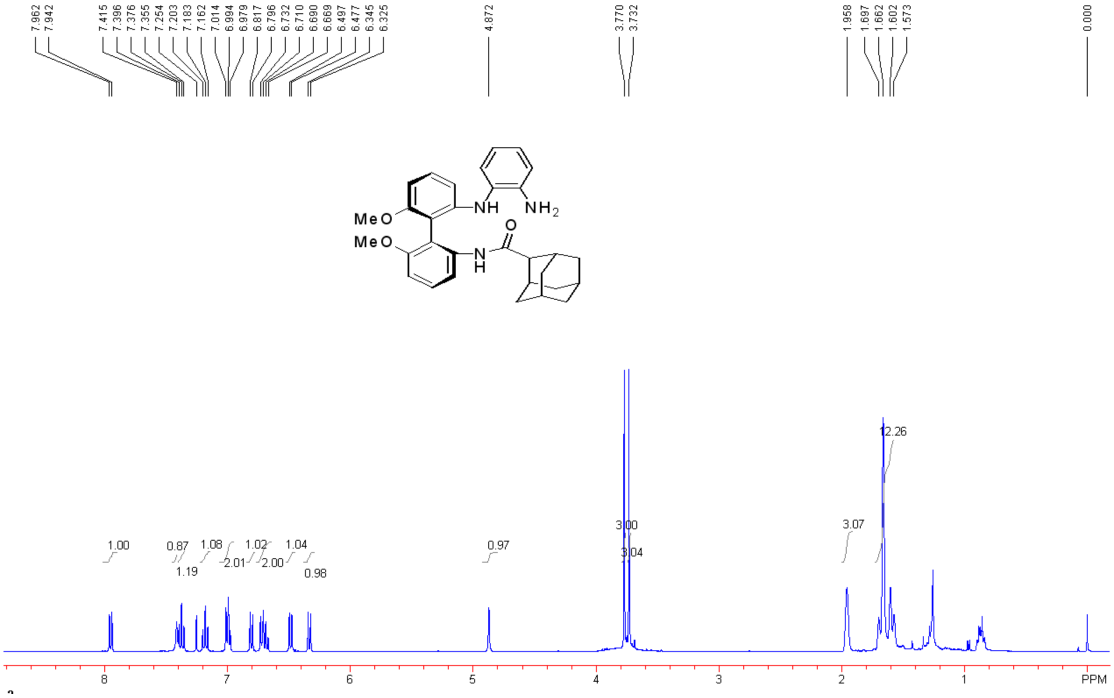

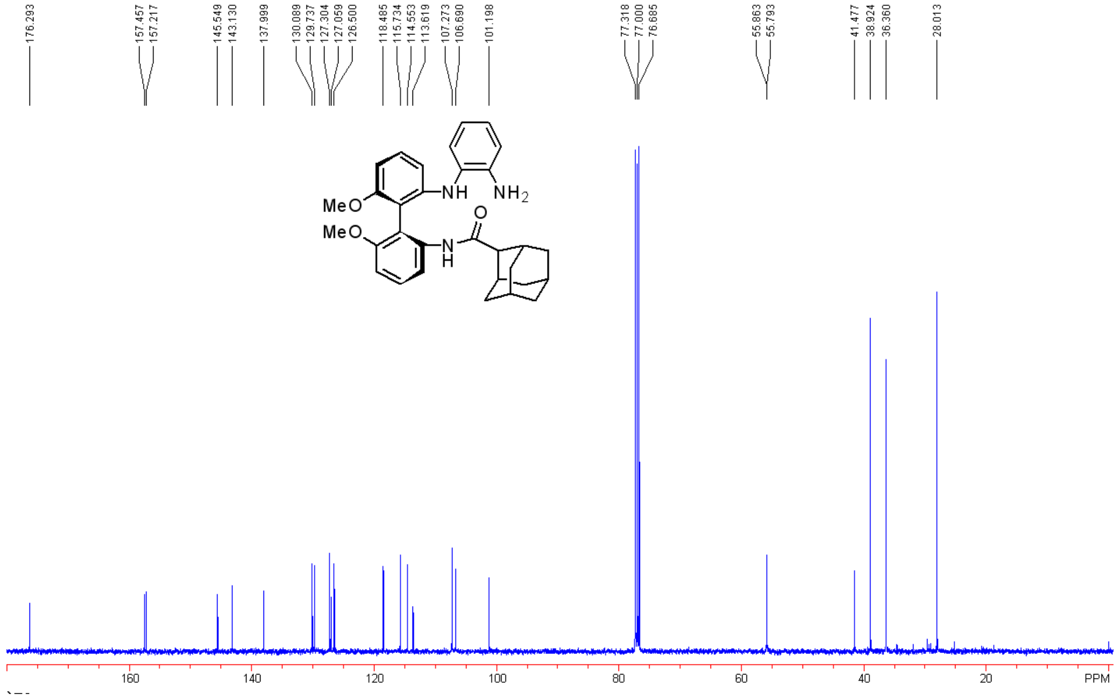

Synthesis of (*S*)-4c

Compound (*S*)-**3c** (248.7 mg, 0.50 mmol) and triethyl orthoformate [$HC(OC_2H_5)_3$] (5.0 mL) containing a small amount of TsOH were heated at 100 °C for 10 h. After excess triethyl orthoformate was removed under reduced pressure, the residue was purified by silica gel flash column chromatography (eluent: petroleum ether/ethyl acetate, 2/3) to give (*S*)-**4c** as a white solid; yield: 223.2 mg (88%). Mp.: 84.8-85.7 °C; $[\alpha]^{20}_{D}$ -42.8 (*c* 0.5, $CHCl_3$); IR (CH_2Cl_2) ν 3427, 2906, 2850, 2683, 1590, 1467, 1431 1258, 1177, 1145, 1087, 1011, 800, 744 cm^{-1}; ^1H NMR (400 MHz, $CDCl_3$, TMS): δ 1.26-1.74 (m, 12H, CH and CH_2), 1.94 (s, 0.7H, OCH_3), 2.00 (brs, 3H, CH), 3.05 (s, 2.3H, OCH_3), 3.89 (s, 3H, OCH_3), 6.40 (d, J = 8.0 Hz, 1H, NH), 7.16-7.25 (m, 5H, ArH), 7.30-7.32 (m, 1H, ArH), 7.40 (s, 1H, CH), 7.59-7.64 (m, 3H, ArH), 7.70-7.72 (m, 1H, ArH); ^{13}C NMR (100 MHz, $CDCl_3$): δ 28.0, 36.4, 39.1, 41.4, 54.9, 55.3, 106.9, 110.1, 111.0, 114.1, 115.7, 119.7, 120.0, 120.1, 122.2, 123.0, 129.9, 130.2, 134.3, 137.2, 137.5, 142.9, 143.2, 156.4, 157.8, 175.9; MS (ESI) *m/e* (%): 508.3 (M^+ + H, 100); HRMS (Micromass LCT) Calcd. for $C_{32}H_{34}N_3O_3$ 508.2600, Found $C_{32}H_{34}N_3O_3$ 508.2607.

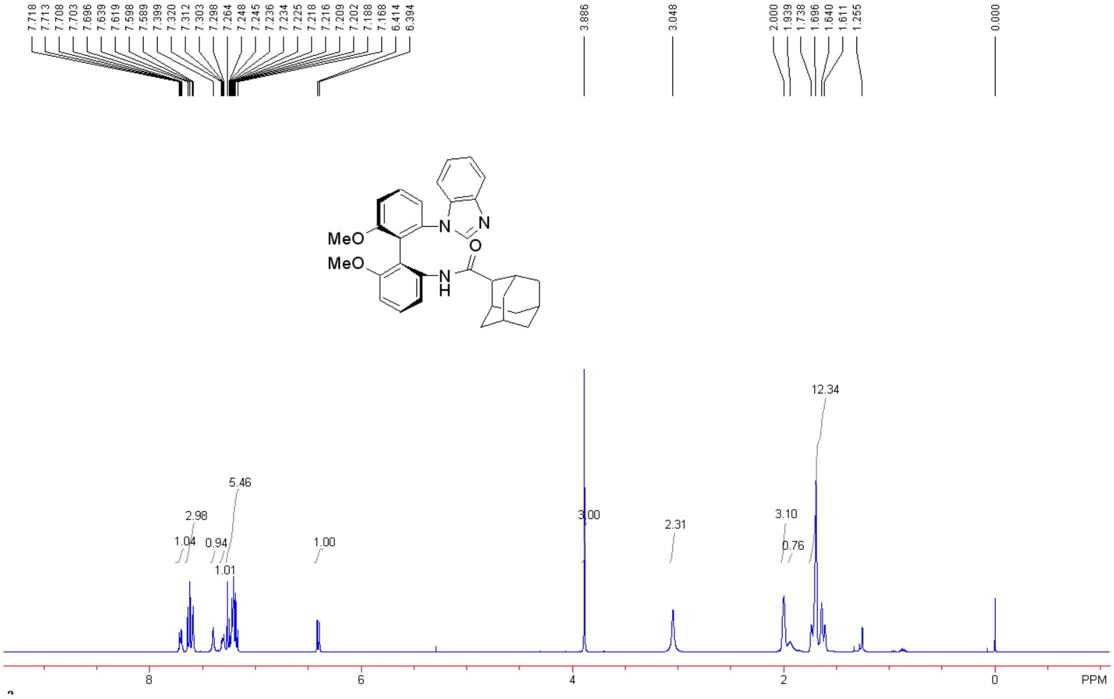

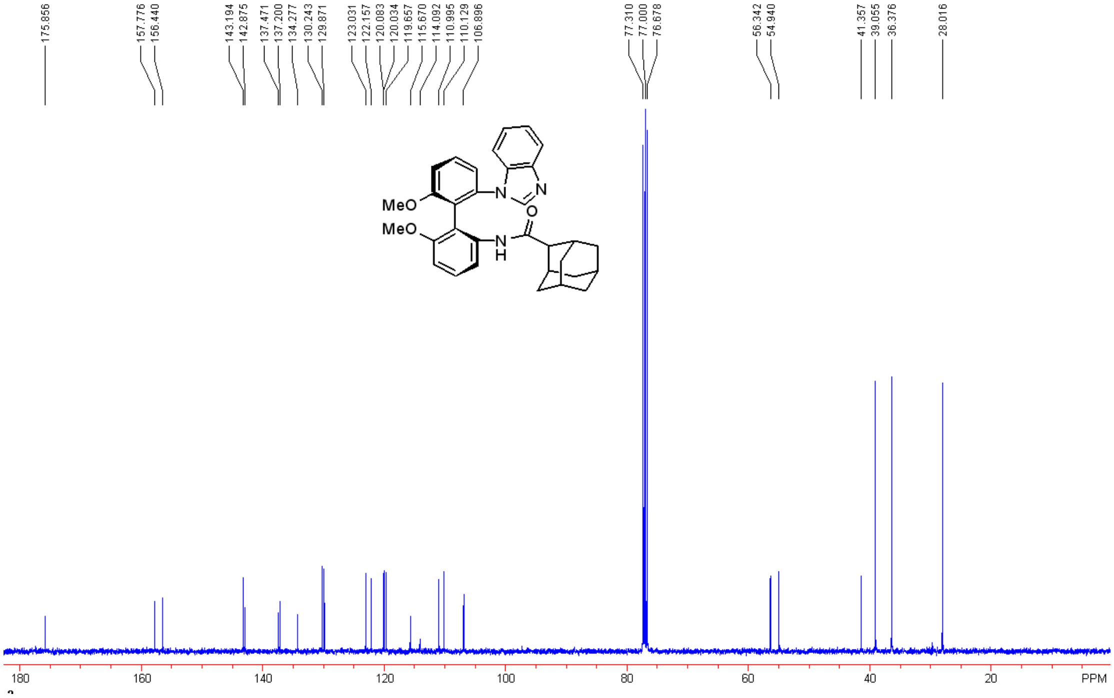

Synthesis of (*S*)-5c

The compound (*S*)-**4c** (253.7 mg, 0.50 mmol) and CH_3I (0.60 mL, 10 mmol) in CH_3CN (4.0 mL) were stirred under reflux for 8 h. After cooling to room temperature, the volatiles were removed under reduced pressure and the solid obtained (*S*)-**5c** was used in the next reaction without further purification. MS (ESI) *m/e*: 522.3 (M^+-I, 100).

Synthesis of NHC-Au(I) complex (*S*)-6c

Compound (*S*)-**5c** (129.8 mg, 0.2 mmol), AuCl·S(Me)$_2$ (58.8 mg, 0.2 mmol), KI (49.8 mg, 0.3 mmol) and ^{t}BuOK (56 mg, 0.5 mmol) were refluxed in THF (10 mL) for 8 h. The volatiles were then removed under reduced pressure and the residue purified by silica gel flash column chromatography (eluent: petroleum ether/ethyl acetate, 2/1-0/1) to give (*S*)-**6c** as white solid (76 mg, 45%). Mp. 161.2-162.8 °C; $[\alpha]^{20}_{D}$ -115.8 (*c* 0.5, $CHCl_3$); IR (CH_2Cl_2) ν 2919, 2850, 1680, 1586, 1466, 1431, 1276, 1257, 1081, 972, 749 cm^{-1}; ^{1}H NMR (400 MHz, $CDCl_3$, TMS): δ 1.67-1.84 (m, 12H, CH and CH_2), 2.06 (brs, 3H, CH), 3.54 (s, OCH_3, 3H), [3.94 (s, OCH_3), 3.96 (s, OCH_3), 3H], [4.071 (s, CH_3), 4.074 (s, CH_3), 3H], 6.24 (d, *J* = 8.4 Hz, 1H, ArH), 6.72 (d, *J* = 8.0 Hz, 1H, ArH), 6.95 (dt, *J* = 8.4, 2.0 Hz, 1H, ArH), 7.10 (dd, *J* = 7.2, 8.4 Hz, 1H, ArH), 7.20-7.26 (m, 2H, ArH), 7.31 (d, *J* = 8.4 Hz, 1H, ArH), 7.42-7.46 (m, 2H, ArH), 7.65 (dt, *J* = 8.0, 1.6 Hz, ArH, 1H), 7.89 (brs, 1H, NH); ^{13}C NMR (100 MHz, $CDCl_3$): δ 28.1, 34.7, 36.4, 39.3, 41.5, 55.6, 56.7, 106.2, 110.2, 112.2, 112.6, 113.7, 115.8, 120.9, 123.0, 124.4, 125.0, 129.5, 130.6, 131.8, 132.7, 137.0, 137.9, 156.0, 156.7, 175.4, 189.0; MS (ESI) *m/e* (%): 718.2 (M^+-I, 100); HRMS (Micromass LCT) Calcd. for $C_{33}H_{35}N_3O_3Au$ 718.2344, Found $C_{33}H_{35}N_3O_3Au$ 718.2316.

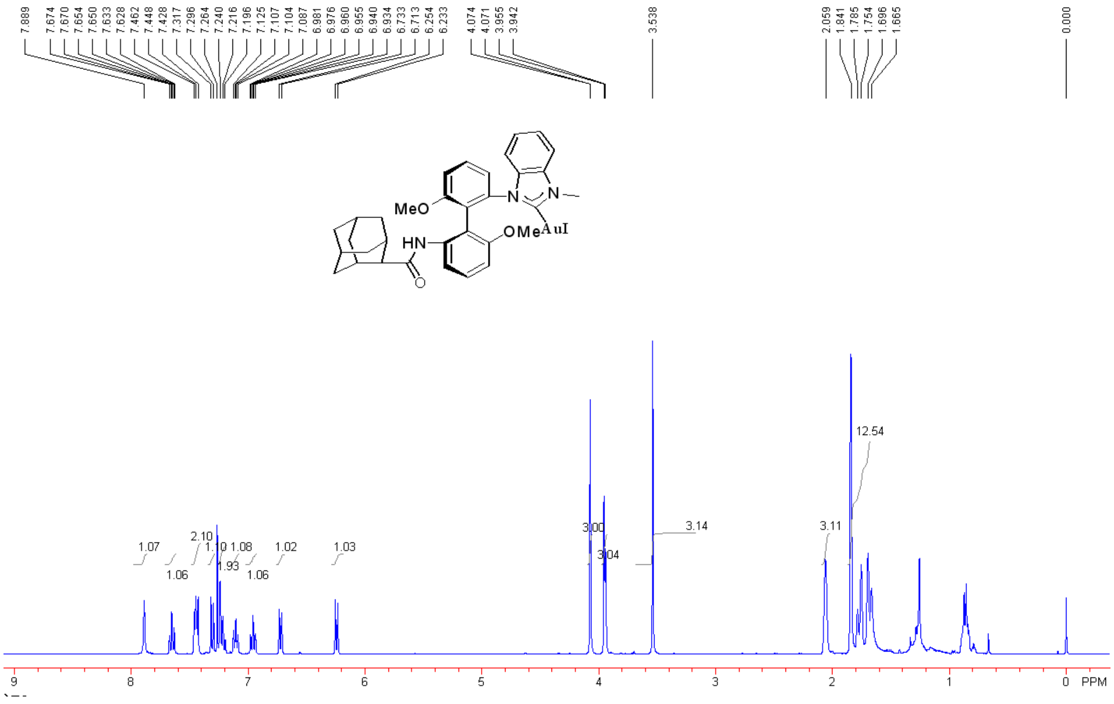

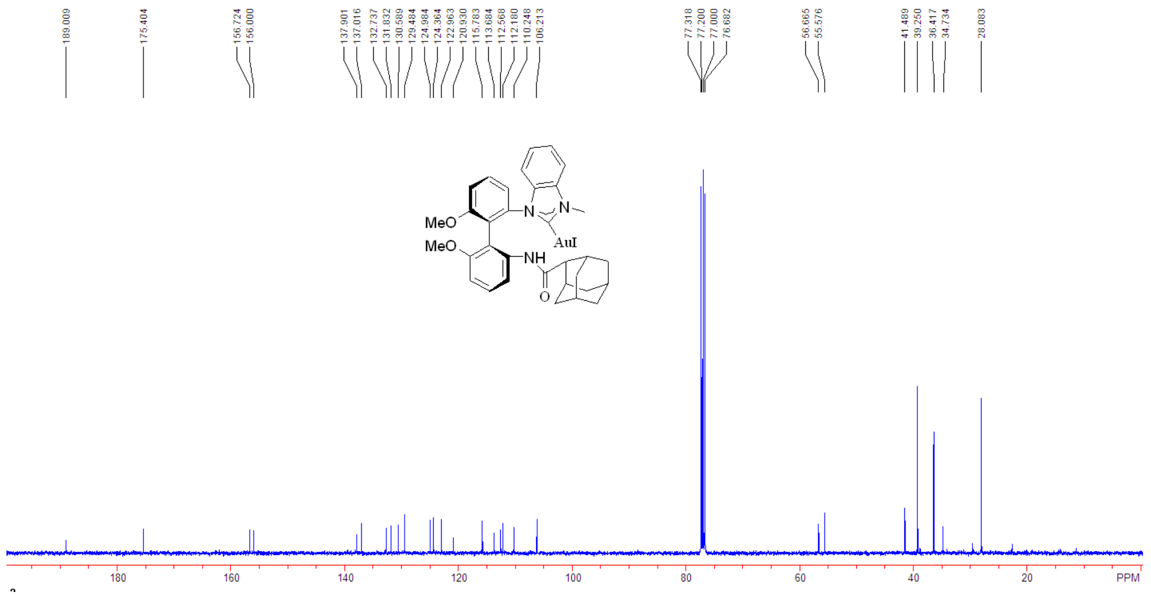

General procedure for the NHC–Pd complex-catalyzed Suzuki–Miyaura cross-coupling reaction of aryl bromides with phenylboronic acids.

The optimized procedure is given below for the reaction given in entry 3 of Table S1. A mixture of NHC-Pd(II) complex **7** (6.8 mg, 0.01 mmol), *t*-BuOK (145.6 mg, 1.3 mmol), 1-bromo-4-methylbenzene (170.0 mg, 1.0 mmol), and phenylboronic acid (220 mg, 1.3 mmol) was dissolved in IPA (2.0 mL). The mixture was stirred at 50 °C for 10 h. The reaction mixture was filtered and then evaporated under vacuum. The residue was purified by column chromatography on silica gel (eluent: petroleum ether) to give **9a** (136.1 mg, 81%) as a white solid.

Table S1. Optimization of NHC–Pd(II) complex **7** catalyzed Suzuki-Miyaura reaction

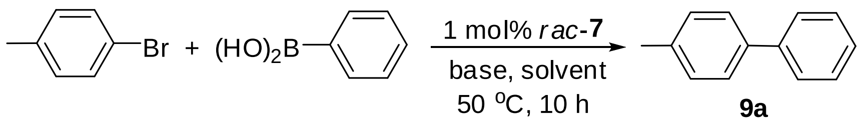

Entry	Solvent	Base	Product	Yield(%)[a]
1	dioxane	KO^tBu	**9a**	37
2	THF	KO^tBu	**9a**	35
3	^iPrOH	KO^tBu	**9a**	83
4	MeCN	KO^tBu	**9a**	68
5	DMF	KO^tBu	**9a**	18
6	^iPrOH	NaO^tBu	**9a**	80
7	^iPrOH	Cs_2CO_3	**9a**	64
8	^iPrOH	$K_3PO_4{\cdot}3H_2O$	**9a**	15
9	^iPrOH	Na_2CHO_3	**9a**	52

[a] Isolated yields

9a: White solid. ^{1}H NMR (400 MHz, $CDCl_3$, TMS): δ 2.38 (s, 3H, CH_3), 7.24 (d, *J* = 5.6 Hz, 2H ArH), 7.32 (t, *J* = 7.2 Hz, 1H, ArH), 7.42 (t, *J* = 7.6 Hz, 2H, ArH), 7.49 (d, *J* = 7.6 Hz, 2H, ArH), 7.58 (d, *J* = 8.8 Hz, 2H, ArH).

9b: White solid. ^{1}H NMR (400 MHz, $CDCl_3$, TMS): δ 2.64 (s, 3H, CH_3), 7.41 (d, *J* = 7.2 Hz, 1H, ArH), 7.47 (t, *J* = 7.2 Hz, 2H, ArH), 7.63 (d, *J* = 8.8 Hz, 2H, ArH), 7.69 (d, *J* = 8.4 Hz, 2H, ArH), 8.03 (d, *J* = 8.4 Hz, 2H, ArH).

9c: White solid. ^{1}H NMR (400 MHz, $CDCl_3$, TMS): δ 7.30-7.32 (m, 2H, ArH), 7.38-7.47 (m, 5H, ArH), 7.56-7.60 (m, 1H, ArH), 7.82 (d, *J* = 8.4 Hz, 1H, ArH).

9d: White solid. ^{1}H NMR (400 MHz, $CDCl_3$, TMS): δ 3.78 (s, 3H, OCH_3), 6.97 (d, *J* = 8.0 Hz, 1H, ArH), 7.02 (t, *J* = 7.6 Hz, 1H, ArH), 7.28-7.32 (m, 3H, ArH), 7.39 (t, *J* = 7.6 Hz, 2H, ArH), 7.52 (d, *J* = 8.4 Hz, 2H, ArH).

9e: Colorless liquid ^{1}H NMR (400 MHz, $CDCl_3$, TMS): δ 2.27 (s, 3H, CH_3), 7.20-7.27 (m, 4H, ArH), 7.31-7.36 (m, 3H, ArH), 7.39-7.43 (m, 2H, ArH).

9f: White solid. ^{1}H NMR (400 MHz, $CDCl_3$, TMS): δ 2.41 (s, 3H, CH_3), 2.63 (s, 3H, CH_3), 7.28 (d, J = 7.6 Hz, 2H, ArH), 7.53 (d, J = 8.4 Hz, 2H, ArH), 7.67 (d, J = 8.4 Hz, 2H, ArH), 8.02 (d, J = 8.4 Hz, 2H, ArH).

9g: White solid. ^{1}H NMR (400 MHz, $CDCl_3$, TMS): δ 2.37 (s, 3H, CH_3), 3.83 (s, 3H, OCH_3), 6.95 (d, J = 8.8 Hz, 2H, ArH), 7.21 (d, J = 8.0 Hz, 2H, ArH), 7.44 (d, J = 8.4 Hz, 2H, ArH), 7.50 (d, J = 8.8 Hz, 2H, ArH).

9h: White solid. ^{1}H NMR (400 MHz, $CDCl_3$, TMS): δ 2.41 (s, 3H, CH_3), 7.23-7.35 (m, 6H, ArH), 7.46 (dd, J = 2.0, 7.6 Hz, 2H, ArH).

General procedure for the Heck–Mizoroki cross-coupling reaction of aryl halide with *n*-butyl acrylate

The optimized procedure is given below for the reaction given in entry 3 of Table S2. Under an argon atmosphere, *n*-butyl acrylate (1.5 mmol), aryl halide (1.0 mmol), Na_2CO_3 (1.1 mmol) and DMAc (2.0 mL) were added successively into a flash-dried Schlenk tube. The reaction mixture was stirred at 140 °C and monitored by TLC (the reaction was usually complete within 18 h). The reaction was quenched with H_2O (15 mL) and extracted with CH_2Cl_2 (3 × 15 mL). The combined organic layers were dried over anhydrous $MgSO_4$ and the solution was concentrated under reduced pressure. Pure products were obtained by flash column chromatography.

Table S2. Optimization of NHC–Pd(II) Complex **7** Catalyzed Heck-Mizoroki Reaction

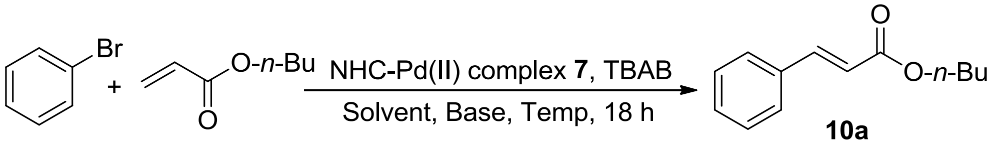

Entry	Solvent	Base	Time (h)	Temp (°C)	product	Yield(%)[a]
1	dioxane	Na_2CO_3	18	100	**10a**	22
2	DMF	Na_2CO_3	18	100	**10a**	50
3	DMAc	Na_2CO_3	18	100	**10a**	70
4	DME	Na_2CO_3	18	100	**10a**	18
5	DMAc	K_2CO_3	18	100	**10a**	43
6	DMAc	KO^tBu	18	100	**10a**	22
7	DMAc	$K_3PO_4 \cdot 3H_2O$	18	100	**10a**	10
8	DMAc	NaOAc	18	100	**10a**	<5
9	DMAc	Na_2CO_3	18	140	**10a**	82

[a] Isolated yields

The ^{1}H NMR spectroscopic data of compounds **10a-e** can be found from the previous report [1].

General procedure for the intramolecular hydroamination reaction catalyzed by NHC–Au(I) complex (*S*)-6a

A mixture of NHC-Au(I) (*S*)-**6a** (7.2 mg, 5 mol%) and AgX (5 mol%) in DCM (0.4 mL) was stirred at room temperature for 5 min and a solution of compound **11** (76.6 mg, 0.20 mmol) in DCM (0.6 mL) added to the resulting solution and the mixture stirred at rt for 36 h. Column chromatography of the reaction mixture gave the desired product. The enantiomeric purity of the product was determined by chiral HPLC analysis.

Compound **11** was prepared according to the previously reported method [2].

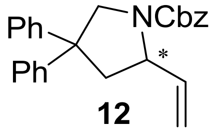

12

^{1}H NMR (25 °C, 400 MHz, 1:1 mixture of rotamers, $CDCl_3$, TMS): δ [2.42 (dd, *J* = 9.6, 18.8 Hz), 2.46 (dd, *J* =10.0, 18.8 Hz), 1:1, 1 H]. 2.82-2.86 (m, 1 H), [3.69 (d, *J* = 9.2 Hz), 3.72 (d, *J* = 8.8 Hz), 1:1, 1 H], 4.08-4.20 (m, 1 H), [4.60 (dd, *J* = 1.2, 11.2 Hz), 4.67 (dd, *J* = 2.0, 11.6 Hz), 1:1, 1 H], 5.04-5.32 (m, 4 H),

5.72-5.83 (m, 1 H), 7.14-7.40 (m, 15 H). $[\alpha]^{20}_{D}$ -8.8 (c 1.0, $CHCl_3$), for 44% ee; Chiralcel AD, hexane/i-PrOH = 75/25, 0.5 mL/min, 230 nm, t_{major} = 22.69 min, t_{minor} = 18.76 min.

WH-500 色 谱 分 析 报 告

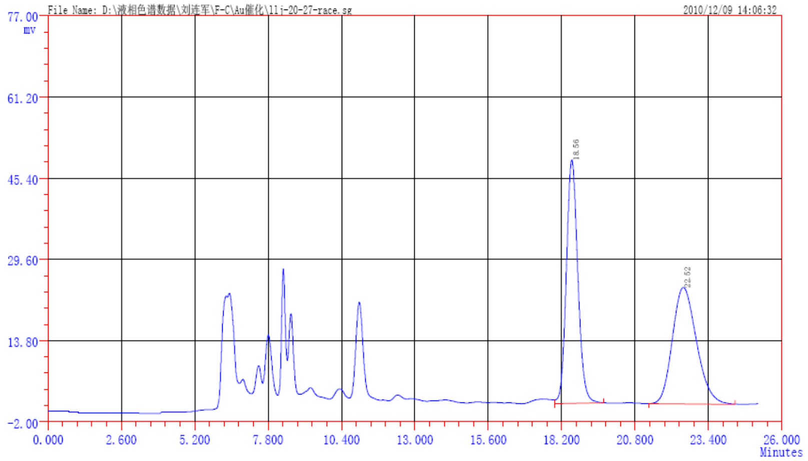

ID	组分名	保留时间	峰高	峰面积	浓度	拖尾因子	理论塔板
1		18.558	47360	1334709.9	49.8315	1.14	8643
2		22.515	22673	1343736.7	50.1685	1.19	2876
	Σ:		70033	2678446.6	100.0000		

WH-500 色 谱 分 析 报 告

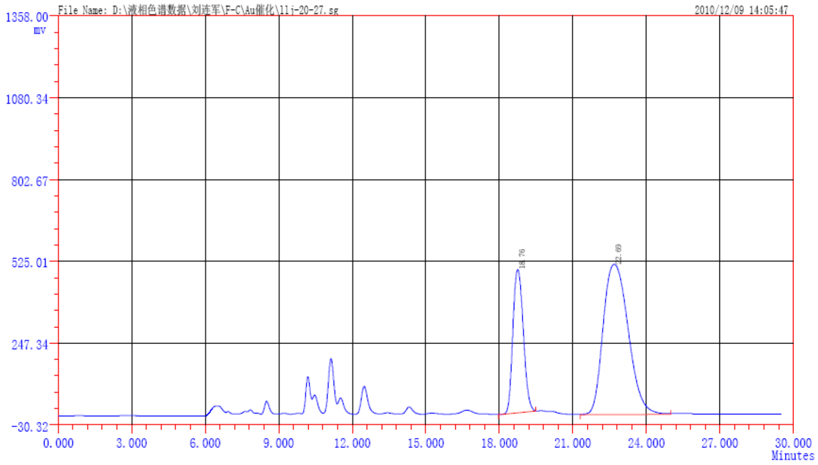

ID	组分名	保留时间	峰高	峰面积	浓度	拖尾因子	理论塔板
1		18.755	486025	14386414.8	28.2768	1.19	8001
2		22.692	510573	36490736.9	71.7232	1.26	2009
	Σ:		996598	50877151.6	100.0000		

References

1. Xu, Q.; Duan, W.-L.; Lei, Z.-Y.; Zhu, Z.-B.; Shi, M. *Tetrahedron* **2005**, *61*, 11225-11229.

2. Zhang, Z.; Bender, C. F.; Widenhoefer, R. A. *Org. Lett.* **2007**, *9*, 2887-2889.
