# Supplementary material for: Synthesis of chiral mono(N-heterocyclic carbene) palladium and gold complexes with a 1,1'-biphenyl scaffold and their applications in catalysis
Source: Beilstein J Org Chem. 2011 May 4;7:555–64. doi: 10.3762/bjoc.7.64 (PMC3107448; doi:10.3762/bjoc.7.64)
Supplement: File 2 — Crystal structure data for NHC–Pd(II) complex 7a. [file Beilstein_J_Org_Chem-07-555-s002.pdf]

Supporting Information

for

Synthesis of chiral mono(N-heterocyclic carbene) palladium and gold complexes with a 1,1'-biphenyl scaffold and their applications in catalysis

Lian-jun Liu[1], Feijun Wang[1], Wenfeng Wang[1], Mei-xin Zhao[1] and Min Shi*[1,2]

Address: [1]Key Laboratory for Advanced Materials and Institute of Fine Chemicals, School of Chemistry & Molecular Engineering, East China University of Science and Technology, 130 MeiLong Road, Shanghai 200237, People's Republic of China and [2]State Key Laboratory of Organometallic Chemistry, Shanghai Institute of Organic Chemistry, Chinese Academy of Sciences, 354 Fenglin Road, Shanghai 200032, People's Republic of China, Fax: 86-21-64166128

Email: Min Shi* - Mshi@mail.sioc.ac.cn

*Corresponding author

Crystal structure data for NHC–Pd(II) complex 7a

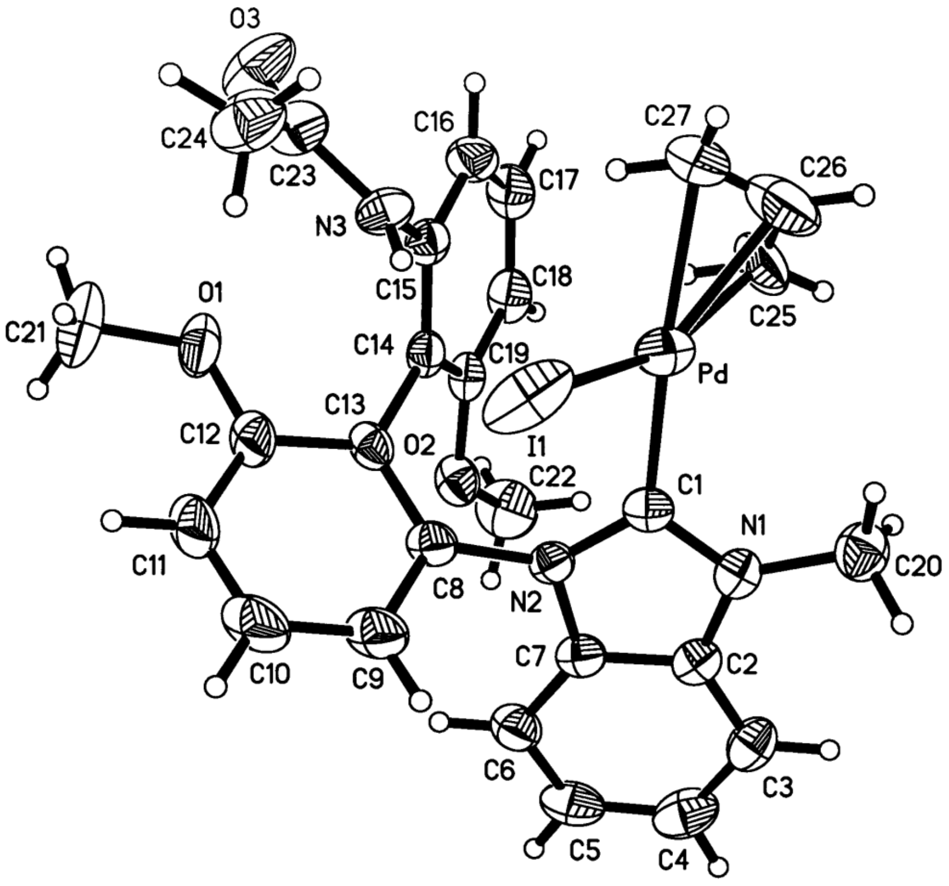

The crystal data of **7a** have been deposited in CCDC with number 754776. Empirical Formula: $C_{27}H_{28}IN_3O_3Pd$; Formula Weight: 675.82; Crystal Color, Habit: colorless, prismatic; Crystal Dimensions: 0.309 x 0.250 x 0.191 mm; Crystal System: Monoclinic; Lattice Type: Primitive; Lattice Parameters: a = 9.8872(8)Å, b = 16.8643(14)Å, c = 16.8744(15)Å, α = 90°, β = 100.713(2)^o, γ = 90°, V = 2764.6(4)Å^3; Space group: P2(1)/c; Z = 4; D_{calc}= 1.624 g/cm^3; F_{000} = 1336; Diffractometer: Rigaku AFC7R; Residuals: R; Rw: 0.0491, 0.1007.

Table 1. Crystal data and structure refinement for cd29547.

Identification code	cd29547
Empirical formula	C27 H28 I N3 O3 Pd
Formula weight	675.82
Temperature	293(2) K
Wavelength	0.71073 A
Crystal system, space group	Monoclinic, P2(1)/c
Unit cell dimensions	a = 9.8872(8) A alpha = 90 deg.<br>b = 16.8643(14) A beta = 100.713(2) deg.<br>c = 16.8744(15) A gamma = 90 deg.
Volume	2764.6(4) A^3
Z, Calculated density	4, 1.624 Mg/m^3
Absorption coefficient	1.819 mm^-1
F(000)	1336
Crystal size	0.309 x 0.250 x 0.191 mm
Theta range for data collection	1.72 to 25.49 deg.
Limiting indices	-11<=h<=11, -20<=k<=14, -18<=l<=20
Reflections collected / unique	14252 / 5145 [R(int) = 0.0528]
Completeness to theta = 25.49	100.0 %
Absorption correction	Empirical
Max. and min. transmission	1.00000 and 0.81653
Refinement method	Full-matrix least-squares on F^2
Data / restraints / parameters	5145 / 1 / 324
Goodness-of-fit on F^2	0.936
Final R indices [I>2sigma(I)]	R1 = 0.0491, wR2 = 0.1007
R indices (all data)	R1 = 0.0780, wR2 = 0.1109
Largest diff. peak and hole	1.093 and -0.564 e.A^-3

Table 2. Atomic coordinates (x 10^4) and equivalent isotropic displacement parameters (A^2 x 10^3) for cd29547.
U(eq) is defined as one third of the trace of the orthogonalized Uij tensor.

	x	y	z	U(eq)
Pd	799(1)	1637(1)	8506(1)	61(1)
I(1)	3189(1)	2293(1)	8413(1)	101(1)
N(1)	-1445(4)	2096(2)	7081(3)	51(1)
N(2)	198(4)	1485(2)	6653(3)	45(1)
N(3)	3215(5)	85(3)	8479(3)	56(1)
O(1)	3786(4)	-485(3)	6955(3)	77(1)
O(2)	-414(3)	-206(2)	6213(2)	59(1)
O(3)	4424(5)	-1028(3)	8889(3)	109(2)
C(1)	-180(5)	1764(3)	7330(3)	47(1)
C(2)	-1864(5)	2021(3)	6261(3)	49(1)
C(3)	-3056(6)	2273(3)	5742(4)	65(2)
C(4)	-3161(7)	2084(4)	4938(5)	80(2)
C(5)	-2141(7)	1654(4)	4659(4)	75(2)
C(6)	-957(6)	1420(3)	5162(4)	63(2)
C(7)	-833(5)	1628(3)	5978(3)	50(1)
C(8)	1526(5)	1204(3)	6548(3)	53(1)
C(9)	2240(6)	1690(4)	6117(4)	75(2)
C(10)	3498(7)	1449(5)	5965(4)	95(2)
C(11)	4052(6)	743(5)	6237(4)	84(2)
C(12)	3336(5)	249(4)	6676(4)	63(2)
C(13)	2065(5)	475(3)	6850(3)	49(1)
C(14)	1342(5)	-67(3)	7336(3)	46(1)
C(15)	1953(5)	-270(3)	8119(3)	49(1)
C(16)	1314(6)	-801(3)	8568(4)	61(2)
C(17)	70(6)	-1125(3)	8212(4)	70(2)
C(18)	-569(6)	-939(3)	7440(4)	62(2)
C(19)	88(5)	-409(3)	6997(3)	50(1)
C(20)	-2246(6)	2498(4)	7603(4)	74(2)
C(21)	5108(7)	-745(5)	6863(5)	119(3)
C(22)	-1763(6)	-434(5)	5853(4)	95(2)
C(23)	4380(6)	-314(4)	8804(4)	74(2)
C(24)	5624(6)	211(4)	9028(4)	96(2)
C(25)	-788(9)	989(5)	8946(5)	100(3)
C(26)	66(16)	1352(7)	9580(7)	158(5)
C(27)	1377(11)	1256(7)	9750(5)	146(5)

Table 3. Bond lengths [A] and angles [deg] for cd29547.

Pd-C(1)	2.050(5)
Pd-C(26)	2.128(9)
Pd-C(25)	2.154(7)
Pd-C(27)	2.168(8)
Pd-I(1)	2.6404(7)
N(1)-C(1)	1.364(6)
N(1)-C(2)	1.373(6)
N(1)-C(20)	1.456(7)
N(2)-C(1)	1.351(6)
N(2)-C(7)	1.400(6)
N(2)-C(8)	1.437(6)
N(3)-C(23)	1.358(7)
N(3)-C(15)	1.415(6)
N(3)-H(3A)	0.819(19)
O(1)-C(12)	1.369(7)
O(1)-C(21)	1.415(7)
O(2)-C(19)	1.368(6)
O(2)-C(22)	1.412(6)
O(3)-C(23)	1.213(7)
C(2)-C(7)	1.374(7)
C(2)-C(3)	1.397(7)
C(3)-C(4)	1.380(8)
C(3)-H(3)	0.9300
C(4)-C(5)	1.393(9)
C(4)-H(4)	0.9300
C(5)-C(6)	1.370(8)
C(5)-H(5)	0.9300
C(6)-C(7)	1.404(7)
C(6)-H(6)	0.9300
C(8)-C(9)	1.375(7)
C(8)-C(13)	1.398(7)
C(9)-C(10)	1.377(9)
C(9)-H(9)	0.9300
C(10)-C(11)	1.354(9)
C(10)-H(10)	0.9300
C(11)-C(12)	1.392(8)
C(11)-H(11)	0.9300
C(12)-C(13)	1.396(7)
C(13)-C(14)	1.495(7)
C(14)-C(19)	1.390(7)
C(14)-C(15)	1.390(7)
C(15)-C(16)	1.399(7)
C(16)-C(17)	1.377(8)
C(16)-H(16)	0.9300
C(17)-C(18)	1.374(8)
C(17)-H(17)	0.9300
C(18)-C(19)	1.399(7)
C(18)-H(18)	0.9300
C(20)-H(20A)	0.9600
C(20)-H(20B)	0.9600
C(20)-H(20C)	0.9600
C(21)-H(21A)	0.9600
C(21)-H(21B)	0.9600
C(21)-H(21C)	0.9600
C(22)-H(22A)	0.9600
C(22)-H(22B)	0.9600
C(22)-H(22C)	0.9600
C(23)-C(24)	1.506(8)
C(24)-H(24A)	0.9600
C(24)-H(24B)	0.9600
C(24)-H(24C)	0.9600
C(25)-C(26)	1.377(14)
C(25)-H(25A)	0.9300
C(25)-H(25B)	0.9300
C(26)-C(27)	1.285(14)
C(26)-H(26)	0.9300
C(27)-H(27A)	0.9300
C(27)-H(27B)	0.9300

C(1)-Pd-C(26)	132.3(5)
C(1)-Pd-C(25)	98.3(3)
C(26)-Pd-C(25)	37.5(4)
C(1)-Pd-C(27)	163.7(4)
C(26)-Pd-C(27)	34.8(4)
C(25)-Pd-C(27)	66.0(4)
C(1)-Pd-I(1)	99.52(13)
C(26)-Pd-I(1)	126.5(5)
C(25)-Pd-I(1)	161.9(3)
C(27)-Pd-I(1)	95.9(3)
C(1)-N(1)-C(2)	111.1(4)
C(1)-N(1)-C(20)	125.1(5)
C(2)-N(1)-C(20)	123.8(5)
C(1)-N(2)-C(7)	110.7(4)
C(1)-N(2)-C(8)	128.4(4)
C(7)-N(2)-C(8)	120.1(4)
C(23)-N(3)-C(15)	125.3(5)
C(23)-N(3)-H(3A)	118(4)
C(15)-N(3)-H(3A)	115(4)
C(12)-O(1)-C(21)	119.3(5)
C(19)-O(2)-C(22)	119.4(4)
N(2)-C(1)-N(1)	105.3(4)
N(2)-C(1)-Pd	128.8(4)
N(1)-C(1)-Pd	125.6(4)
N(1)-C(2)-C(7)	106.9(5)
N(1)-C(2)-C(3)	131.6(5)
C(7)-C(2)-C(3)	121.5(6)
C(4)-C(3)-C(2)	116.6(6)
C(4)-C(3)-H(3)	121.7
C(2)-C(3)-H(3)	121.7
C(3)-C(4)-C(5)	121.7(6)
C(3)-C(4)-H(4)	119.2
C(5)-C(4)-H(4)	119.1
C(6)-C(5)-C(4)	122.0(6)
C(6)-C(5)-H(5)	119.0
C(4)-C(5)-H(5)	119.0
C(5)-C(6)-C(7)	116.4(6)
C(5)-C(6)-H(6)	121.8
C(7)-C(6)-H(6)	121.8
C(2)-C(7)-N(2)	106.0(5)
C(2)-C(7)-C(6)	121.7(5)
N(2)-C(7)-C(6)	132.2(5)
C(9)-C(8)-C(13)	121.2(5)
C(9)-C(8)-N(2)	116.1(5)
C(13)-C(8)-N(2)	122.7(4)
C(10)-C(9)-C(8)	119.6(6)
C(10)-C(9)-H(9)	120.2
C(8)-C(9)-H(9)	120.2
C(11)-C(10)-C(9)	121.3(6)
C(11)-C(10)-H(10)	119.4
C(9)-C(10)-H(10)	119.4
C(10)-C(11)-C(12)	119.5(6)
C(10)-C(11)-H(11)	120.2
C(12)-C(11)-H(11)	120.2
O(1)-C(12)-C(11)	124.1(5)
O(1)-C(12)-C(13)	114.9(5)
C(11)-C(12)-C(13)	121.0(6)
C(12)-C(13)-C(8)	117.4(5)
C(12)-C(13)-C(14)	119.3(5)
C(8)-C(13)-C(14)	123.2(4)
C(19)-C(14)-C(15)	119.1(5)
C(19)-C(14)-C(13)	120.6(5)
C(15)-C(14)-C(13)	120.2(4)
C(14)-C(15)-C(16)	120.8(5)
C(14)-C(15)-N(3)	119.8(4)
C(16)-C(15)-N(3)	119.3(5)
C(17)-C(16)-C(15)	118.3(6)
C(17)-C(16)-H(16)	120.9
C(15)-C(16)-H(16)	120.9
C(18)-C(17)-C(16)	122.7(5)
C(18)-C(17)-H(17)	118.7

C(16)-C(17)-H(17)	118.7
C(17)-C(18)-C(19)	118.3(5)
C(17)-C(18)-H(18)	120.8
C(19)-C(18)-H(18)	120.8
O(2)-C(19)-C(14)	115.4(5)
O(2)-C(19)-C(18)	123.8(5)
C(14)-C(19)-C(18)	120.8(5)
N(1)-C(20)-H(20A)	109.5
N(1)-C(20)-H(20B)	109.5
H(20A)-C(20)-H(20B)	109.5
N(1)-C(20)-H(20C)	109.5
H(20A)-C(20)-H(20C)	109.5
H(20B)-C(20)-H(20C)	109.5
O(1)-C(21)-H(21A)	109.5
O(1)-C(21)-H(21B)	109.5
H(21A)-C(21)-H(21B)	109.5
O(1)-C(21)-H(21C)	109.5
H(21A)-C(21)-H(21C)	109.5
H(21B)-C(21)-H(21C)	109.5
O(2)-C(22)-H(22A)	109.5
O(2)-C(22)-H(22B)	109.5
H(22A)-C(22)-H(22B)	109.5
O(2)-C(22)-H(22C)	109.5
H(22A)-C(22)-H(22C)	109.5
H(22B)-C(22)-H(22C)	109.5
O(3)-C(23)-N(3)	123.3(6)
O(3)-C(23)-C(24)	123.0(6)
N(3)-C(23)-C(24)	113.7(6)
C(23)-C(24)-H(24A)	109.5
C(23)-C(24)-H(24B)	109.5
H(24A)-C(24)-H(24B)	109.5
C(23)-C(24)-H(24C)	109.5
H(24A)-C(24)-H(24C)	109.5
H(24B)-C(24)-H(24C)	109.5
C(26)-C(25)-Pd	70.2(5)
C(26)-C(25)-H(25A)	120.0
Pd-C(25)-H(25A)	73.2
C(26)-C(25)-H(25B)	120.0
Pd-C(25)-H(25B)	128.9
H(25A)-C(25)-H(25B)	120.0
C(27)-C(26)-C(25)	124.4(12)
C(27)-C(26)-Pd	74.3(6)
C(25)-C(26)-Pd	72.3(5)
C(27)-C(26)-H(26)	117.8
C(25)-C(26)-H(26)	117.8
Pd-C(26)-H(26)	128.0
C(26)-C(27)-Pd	70.9(6)
C(26)-C(27)-H(27A)	120.0
Pd-C(27)-H(27A)	72.1
C(26)-C(27)-H(27B)	120.0
Pd-C(27)-H(27B)	129.4
H(27A)-C(27)-H(27B)	120.0

Symmetry transformations used to generate equivalent atoms:

Table 4. Anisotropic displacement parameters (A^2 x 10^3) for cd29547.
The anisotropic displacement factor exponent takes the form:
-2 pi^2 [h^2 a*^2 U11 + ... + 2 h k a* b* U12]

	U11	U22	U33	U23	U13	U12
Pd	81(1)	44(1)	51(1)	-4(1)	-2(1)	-1(1)
I(1)	78(1)	50(1)	152(1)	10(1)	-40(1)	-18(1)
N(1)	48(3)	44(2)	60(3)	-4(2)	8(2)	-3(2)
N(2)	38(2)	46(2)	53(3)	9(2)	9(2)	-2(2)
N(3)	62(3)	42(3)	60(3)	5(2)	-1(2)	-2(2)
O(1)	51(2)	90(3)	94(3)	-6(3)	22(2)	19(2)
O(2)	40(2)	74(3)	62(3)	-7(2)	10(2)	-9(2)
O(3)	84(3)	74(3)	159(5)	39(3)	-1(3)	13(3)
C(1)	46(3)	41(3)	52(3)	2(2)	7(3)	-13(2)
C(2)	43(3)	42(3)	60(4)	3(3)	4(3)	-9(2)
C(3)	51(4)	59(4)	81(5)	0(3)	5(3)	4(3)
C(4)	61(4)	83(5)	84(6)	18(4)	-19(4)	-6(3)
C(5)	84(5)	89(5)	46(4)	12(3)	-4(3)	-16(4)
C(6)	64(4)	75(4)	51(4)	3(3)	12(3)	-1(3)
C(7)	43(3)	53(3)	53(4)	8(3)	3(3)	-7(2)
C(8)	39(3)	62(3)	57(4)	14(3)	9(3)	-10(2)
C(9)	50(4)	96(5)	79(5)	31(4)	11(3)	-14(3)
C(10)	58(4)	141(7)	88(6)	41(5)	18(4)	-27(4)
C(11)	39(3)	133(7)	86(5)	13(5)	24(3)	-2(4)
C(12)	33(3)	90(5)	67(4)	0(3)	9(3)	-3(3)
C(13)	37(3)	65(4)	47(3)	-2(3)	11(2)	-9(2)
C(14)	39(3)	42(3)	59(4)	0(2)	18(3)	3(2)
C(15)	52(3)	37(3)	60(4)	2(2)	15(3)	2(2)
C(16)	77(4)	47(3)	61(4)	9(3)	18(3)	0(3)
C(17)	75(4)	50(3)	95(6)	9(3)	40(4)	-5(3)
C(18)	57(4)	47(3)	88(5)	-2(3)	27(4)	-5(3)
C(19)	44(3)	48(3)	60(4)	-7(3)	17(3)	5(2)
C(20)	79(4)	66(4)	76(5)	-8(3)	15(4)	3(3)
C(21)	65(5)	130(7)	165(9)	-20(6)	30(5)	35(4)
C(22)	51(4)	130(6)	97(6)	6(5)	-2(4)	-24(4)
C(23)	68(4)	70(4)	78(5)	13(4)	3(3)	-1(4)
C(24)	72(5)	100(6)	105(6)	17(4)	-12(4)	-7(4)
C(25)	119(7)	110(6)	92(7)	20(5)	75(6)	10(5)
C(26)	258(16)	154(10)	84(8)	41(7)	91(10)	49(11)
C(27)	189(11)	203(11)	57(6)	42(6)	47(7)	105(9)

Table 5. Hydrogen coordinates (x 10^4) and isotropic displacement parameters (A^2 x 10^3) for cd29547.

	x	y	z	U(eq)
H(3)	-3743	2553	5930	78
H(4)	-3933	2248	4572	96
H(5)	-2267	1522	4115	91
H(6)	-273	1138	4974	76
H(9)	1876	2178	5928	90
H(10)	3976	1777	5670	114
H(11)	4905	590	6132	101
H(16)	1719	-933	9094	73
H(17)	-354	-1484	8505	84
H(18)	-1417	-1159	7218	75
H(20A)	-2543	3004	7376	111
H(20B)	-1687	2571	8127	111
H(20C)	-3035	2182	7650	111
H(21A)	5152	-766	6300	179
H(21B)	5278	-1264	7094	179
H(21C)	5791	-383	7132	179
H(22A)	-1846	-1000	5880	142
H(22B)	-1949	-268	5299	142
H(22C)	-2411	-190	6136	142
H(24A)	6444	-100	9053	144
H(24B)	5621	449	9545	144
H(24C)	5600	620	8629	144
H(25A)	-423	648	8607	120
H(25B)	-1731	1085	8856	120
H(26)	-333	1690	9907	189
H(27A)	1808	922	9435	176
H(27B)	1894	1519	10188	176
H(3A)	3280(50)	560(13)	8390(30)	55(16)

Table 6. Torsion angles [deg] for cd29547.

C(7)-N(2)-C(1)-N(1)	-1.0(5)
C(8)-N(2)-C(1)-N(1)	168.3(4)
C(7)-N(2)-C(1)-Pd	173.4(3)
C(8)-N(2)-C(1)-Pd	-17.3(7)
C(2)-N(1)-C(1)-N(2)	0.7(5)
C(20)-N(1)-C(1)-N(2)	-177.9(4)
C(2)-N(1)-C(1)-Pd	-174.0(3)
C(20)-N(1)-C(1)-Pd	7.4(7)
C(26)-Pd-C(1)-N(2)	-135.6(6)
C(25)-Pd-C(1)-N(2)	-117.8(4)
C(27)-Pd-C(1)-N(2)	-103.1(10)
I(1)-Pd-C(1)-N(2)	58.7(4)
C(26)-Pd-C(1)-N(1)	37.8(6)
C(25)-Pd-C(1)-N(1)	55.6(4)
C(27)-Pd-C(1)-N(1)	70.3(11)
I(1)-Pd-C(1)-N(1)	-127.9(4)
C(1)-N(1)-C(2)-C(7)	0.0(5)
C(20)-N(1)-C(2)-C(7)	178.6(5)
C(1)-N(1)-C(2)-C(3)	-179.2(5)
C(20)-N(1)-C(2)-C(3)	-0.5(8)
N(1)-C(2)-C(3)-C(4)	-179.0(5)
C(7)-C(2)-C(3)-C(4)	2.0(8)
C(2)-C(3)-C(4)-C(5)	0.9(9)
C(3)-C(4)-C(5)-C(6)	-2.3(10)
C(4)-C(5)-C(6)-C(7)	0.7(9)
N(1)-C(2)-C(7)-N(2)	-0.6(5)
C(3)-C(2)-C(7)-N(2)	178.6(4)
N(1)-C(2)-C(7)-C(6)	177.1(5)
C(3)-C(2)-C(7)-C(6)	-3.7(8)
C(1)-N(2)-C(7)-C(2)	1.1(5)
C(8)-N(2)-C(7)-C(2)	-169.3(4)
C(1)-N(2)-C(7)-C(6)	-176.3(5)
C(8)-N(2)-C(7)-C(6)	13.4(8)
C(5)-C(6)-C(7)-C(2)	2.2(8)
C(5)-C(6)-C(7)-N(2)	179.2(5)
C(1)-N(2)-C(8)-C(9)	-108.1(6)
C(7)-N(2)-C(8)-C(9)	60.4(6)
C(1)-N(2)-C(8)-C(13)	73.1(7)
C(7)-N(2)-C(8)-C(13)	-118.5(5)
C(13)-C(8)-C(9)-C(10)	1.2(9)
N(2)-C(8)-C(9)-C(10)	-177.6(6)
C(8)-C(9)-C(10)-C(11)	-0.4(11)
C(9)-C(10)-C(11)-C(12)	0.3(12)
C(21)-O(1)-C(12)-C(11)	5.7(9)
C(21)-O(1)-C(12)-C(13)	-175.3(6)
C(10)-C(11)-C(12)-O(1)	177.9(6)
C(10)-C(11)-C(12)-C(13)	-1.1(10)
O(1)-C(12)-C(13)-C(8)	-177.2(5)
C(11)-C(12)-C(13)-C(8)	1.8(8)
O(1)-C(12)-C(13)-C(14)	2.1(8)
C(11)-C(12)-C(13)-C(14)	-178.9(6)
C(9)-C(8)-C(13)-C(12)	-1.9(8)
N(2)-C(8)-C(13)-C(12)	176.8(5)
C(9)-C(8)-C(13)-C(14)	178.8(5)
N(2)-C(8)-C(13)-C(14)	-2.4(8)
C(12)-C(13)-C(14)-C(19)	-115.1(6)
C(8)-C(13)-C(14)-C(19)	64.1(7)
C(12)-C(13)-C(14)-C(15)	61.2(7)
C(8)-C(13)-C(14)-C(15)	-119.5(6)
C(19)-C(14)-C(15)-C(16)	-0.9(7)
C(13)-C(14)-C(15)-C(16)	-177.4(4)
C(19)-C(14)-C(15)-N(3)	-178.7(4)
C(13)-C(14)-C(15)-N(3)	4.8(7)
C(23)-N(3)-C(15)-C(14)	-124.9(6)
C(23)-N(3)-C(15)-C(16)	57.3(7)
C(14)-C(15)-C(16)-C(17)	0.7(7)
N(3)-C(15)-C(16)-C(17)	178.5(5)
C(15)-C(16)-C(17)-C(18)	-0.8(8)

C(16)-C(17)-C(18)-C(19)	1.1(8)
C(22)-O(2)-C(19)-C(14)	-171.3(5)
C(22)-O(2)-C(19)-C(18)	10.3(7)
C(15)-C(14)-C(19)-O(2)	-177.2(4)
C(13)-C(14)-C(19)-O(2)	-0.7(6)
C(15)-C(14)-C(19)-C(18)	1.3(7)
C(13)-C(14)-C(19)-C(18)	177.7(4)
C(17)-C(18)-C(19)-O(2)	176.9(5)
C(17)-C(18)-C(19)-C(14)	-1.4(8)
C(15)-N(3)-C(23)-O(3)	-7.2(10)
C(15)-N(3)-C(23)-C(24)	171.0(5)
C(1)-Pd-C(25)-C(26)	-158.2(7)
C(27)-Pd-C(25)-C(26)	26.2(7)
I(1)-Pd-C(25)-C(26)	33.1(11)
Pd-C(25)-C(26)-C(27)	-55.7(10)
C(1)-Pd-C(26)-C(27)	164.7(7)
C(25)-Pd-C(26)-C(27)	134.9(12)
I(1)-Pd-C(26)-C(27)	-32.9(10)
C(1)-Pd-C(26)-C(25)	29.8(8)
C(27)-Pd-C(26)-C(25)	-134.9(12)
I(1)-Pd-C(26)-C(25)	-167.8(4)
C(25)-C(26)-C(27)-Pd	54.8(10)
C(1)-Pd-C(27)-C(26)	-44.1(15)
C(25)-Pd-C(27)-C(26)	-28.2(8)
I(1)-Pd-C(27)-C(26)	154.0(8)

Symmetry transformations used to generate equivalent atoms:

Table 7. Hydrogen bonds for cd29547 [A and deg.].

D-H...A	d(D-H)	d(H...A)	d(D...A)	<(DHA)
C(3)-H(3)...O(3)#1	0.93	2.52	3.278(7)	138.8
C(21)-H(21B)...I(1)#2	0.96	3.07	3.780(7)	131.8
N(3)-H(3A)...I(1)	0.819(19)	2.92(2)	3.725(5)	167(4)

Symmetry transformations used to generate equivalent atoms:
#1 -x,y+1/2,-z+3/2 #2 -x+1,y-1/2,-z+3/2
