# Supplementary material for: Synthesis of chiral mono(N-heterocyclic carbene) palladium and gold complexes with a 1,1'-biphenyl scaffold and their applications in catalysis
Source: Beilstein J Org Chem. 2011 May 4;7:555–64. doi: 10.3762/bjoc.7.64 (PMC3107448; doi:10.3762/bjoc.7.64)
Supplement: File 3 — Crystal structure data for NHC–Au(I) complex 6a. [file Beilstein_J_Org_Chem-07-555-s003.pdf]

Supporting Information

for

Synthesis of chiral mono(N-heterocyclic carbene) palladium and gold complexes with a 1,1'-biphenyl scaffold and their applications in catalysis

Lian-jun Liu[1], Feijun Wang[1], Wenfeng Wang[1], Mei-xin Zhao[1] and Min Shi*[1,2]

Address: [1]Key Laboratory for Advanced Materials and Institute of Fine Chemicals, School of Chemistry & Molecular Engineering, East China University of Science and Technology, 130 MeiLong Road, Shanghai 200237, People's Republic of China and [2]State Key Laboratory of Organometallic Chemistry, Shanghai Institute of Organic Chemistry, Chinese Academy of Sciences, 354 Fenglin Road, Shanghai 200032, People's Republic of China, Fax: 86-21-64166128

Email: Min Shi* - Mshi@mail.sioc.ac.cn

*Corresponding author

Crystal structure data for NHC–Au(I) complex 6a

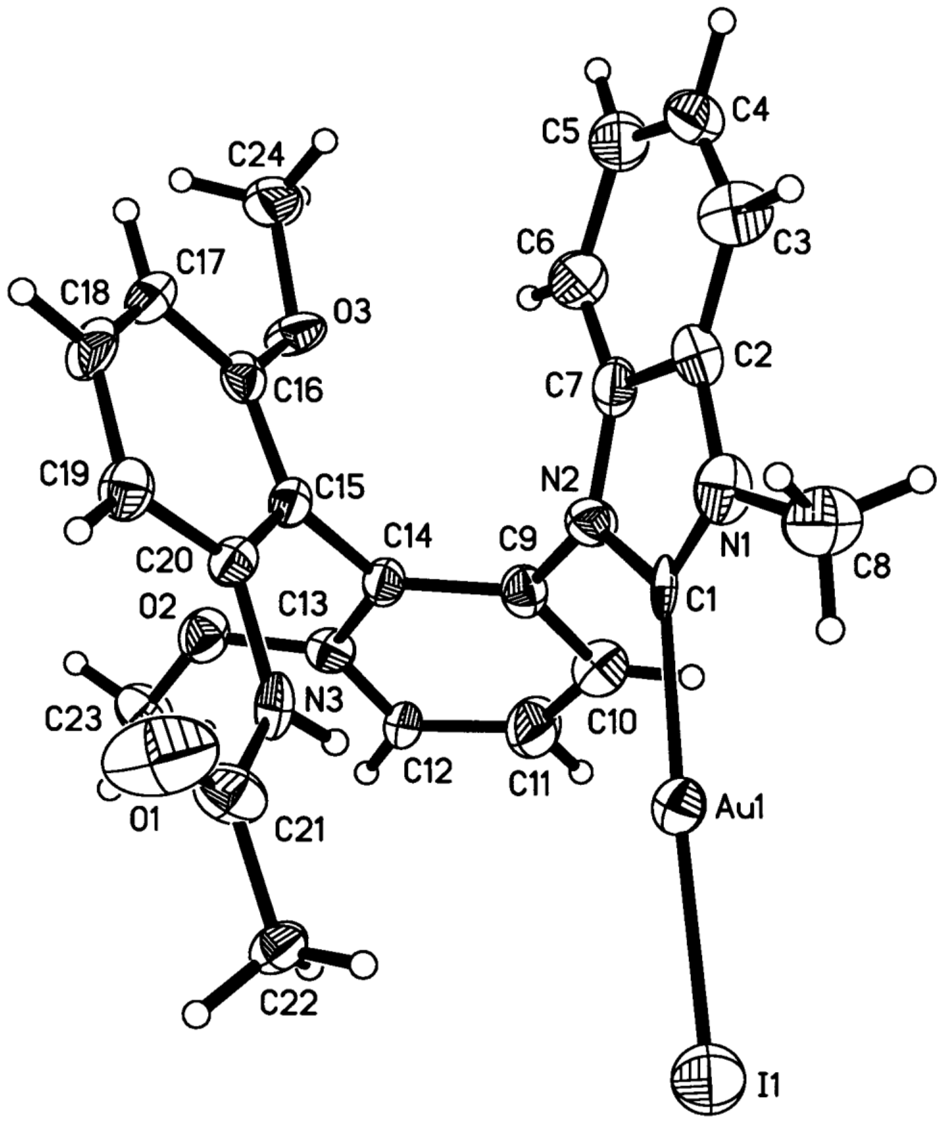

The crystal data of **6a** have been deposited in CCDC with number 782844. Empirical Formula: $C_{24}H_{23}AuIN_3O_3$; Formula Weight: 725.32; Crystal Color, Habit: colorless, prismatic; Crystal Dimensions: 0.277 x 0.268 x 0.224 mm; Crystal System: Monoclinic; Lattice Type: Primitive; Lattice Parameters: a = 9.2786(7)Å, b = 25.5371(19)Å, c = 10.1633(7)Å, α = 90°, β = 104.9480(10)°, γ = 90°, V = 2326.7(3)Å^3; Space group: P2(1); Z = 4; D_{calc}= 2.071 g/cm^3; F_{000} = 1376; Diffractometer: Rigaku AFC7R; Residuals: R; Rw: 0.0478, 0.1273.
